# Lithium-selective supramolecular assembly and capture by tripeptide gelators

**DOI:** 10.1039/d6sc01183g

**Published:** 2026-04-13

**Authors:** Dipankar Ghosh, Ralf Schweins, Andrew J. Smith, Dave J. Adams

**Affiliations:** a School of Chemistry, University of Glasgow Glasgow G12 8QQ UK dave.adams@glasgow.ac.uk; b Large Scale Structures Group, Institut Laue-Langevin 71 Avenue des Martyrs, CS 20156 F-38042 Grenoble CEDEX 9 France; c Diamond Light Source Ltd Diamond House, Harwell Science and Innovation Campus Didcot Oxfordshire OX11 0DE UK

## Abstract

We report a simple design strategy to introduce lithium-responsiveness into N-capped peptide low-molecular-weight gelators by incorporating the FFD tripeptide motif (FF extended with Asp). Asp adds an oxygen-rich carboxylate residue that enables cation-mediated assembly. In high pH aqueous solutions, 2NapFFD shows a pronounced cation selectivity. Li^+^ generates highly viscous, shear-thinning solutions with birefringent textures, while other Group 1 metals and bulky organic counterions result in low-viscosity and weakly ordered solutions. SAXS and SANS reveal that the addition of Li^+^ produces substantially extended micellar structures consistent with long cylindrical assemblies, whereas other monovalent cations lead to the formation of short cylindrical objects. The Li^+^ selectivity is intrinsic to the FFD sequence, with other aromatic caps tuning packing and mesoscale order. Finally, dialysis-driven Li^+^ exchange induces gelation and enables us to quantify the Li^+^ uptake by ICP-OES, illustrating potential for selective lithium capture.

## Abstract 2

LMWGs containing the tripeptide FFD motif show selective Li^+^ responsiveness, forming entangled networks in the presence of Li^+^. This offers a simple supramolecular route to selective Li^+^ uptake over competing monovalent cations.

## Graphical abstract

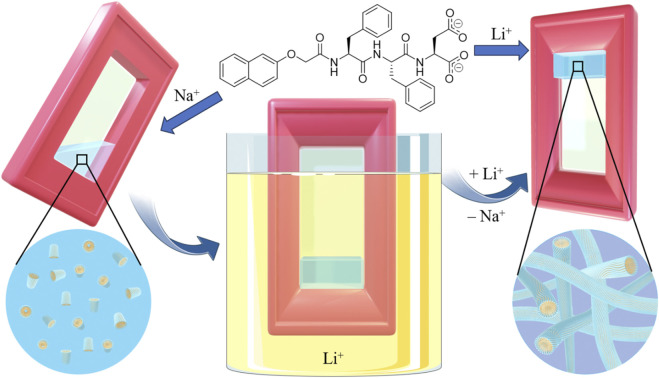

## Introduction

Peptide-based low molecular weight gelators (LMWGs) have emerged as versatile building blocks for supramolecular materials due to their inherent biocompatibility, modular design, and capacity to self-assemble through non-covalent interactions.^1–3^ Despite their structural simplicity, they can adopt a wide range of morphologies, including micelles, fibres, tapes, and gels.^4–7^ These features underpin their growing utility in applications such as drug delivery, tissue engineering, and stimuli-responsive materials.^8–10^ Among the dipeptide gelators, systems incorporating diphenylalanine (FF) motif are the most extensively studied as they readily form robust supramolecular assemblies driven by cooperative aromatic π–π interactions and peptide–peptide hydrogen bonding,^1,11,12^ exemplified by 2NapFF and 1ThNapFF.^4,13,14^ The dipeptide 2NapFF, which incorporates a 2-naphthoxyacetic acid cap and two phenylalanine residues, assembles into tubular micelles that form supramolecular gels.^4,13,15^ 1ThNapFF similarly forms wormlike micelles under basic conditions, yielding shear-thinning solutions.^11,14^ These examples highlight how small changes in molecular design can profoundly influence the resulting self-assembled structures and material behaviour. Such systems exemplify how peptide LMWGs can be rationally tuned for specific structural and functional outcomes.^11^

Building on these strategies, we focused on gelators incorporating the tripeptide FFD motif, obtained by extending the FF sequence with an aspartic acid residue. The FFD motif can be paired with different N-terminal aromatic caps, providing a modular route to tune assembly and metal-ion response. This modification retains the hydrophobic and aromatic character of the diphenylalanine core that supports self-assembly, while introducing an additional carboxylate functionality through the aspartic acid side chain. The added carboxylate can act as a coordination site for metal ions, providing a handle to introduce ion-responsiveness.^16,17^

Metal ions are well known to modulate peptide self-assembly by screening charges, stabilizing specific conformations, or directly coordinating with side-chain functionalities.^17–22^ The identity of the cation plays a crucial role. Different cations can prompt distinct effects on assembly, morphology, and mechanical properties.^19,21,22^ Harnessing cation-specific interactions is therefore an attractive strategy for designing selective ion-responsive materials, with potential applications in sensing, separation, and adaptive materials.^17,22,23^ Several amino acid residues are known to participate in metal ion coordination. For example, histidine binds transition metals such as Zn^2+^, Cu^2+^, and Ni^2+^*via* its imidazole group;^24–26^ aspartate and glutamate commonly coordinate Ca^2+^ and Mg^2+^;^27,28^ cysteine binds soft metals like Hg^2+^ and Cd^2+^ through thiol;^29,30^ and tyrosine can bind Fe^3+^ and Al^3+^ under appropriate conditions.^31^ Aspartic acid, with its side-chain carboxylate, exhibits a particularly strong affinity for small, hard metal ions such as lithium.^32–34^ Its geometry and electron density make it ideally suited to coordinate with Li^+^, which strongly favours oxygen donors in rigid coordination environments.^23,35^ This preference is supported by structural studies of lithium-aspartate complexes, motivating the incorporation of aspartic acid into ion-responsive peptides.^34^

The rational introduction of aspartic acid into a self-assembling peptide thus presents a promising approach to creating lithium-responsive materials.^17,32,34^ Given the rising demand for lithium in energy storage technologies and increasing interest in lithium separation from aqueous environments, there is strong motivation to develop supramolecular systems capable of recognizing and selectively interacting with lithium ions.^23,36^ Peptides provide a biodegradable and tunable scaffold for such applications, offering advantages in sustainability and design flexibility.^37,38^ Here, we present the design of gelators containing the tripeptide FFD moiety as a cation-responsive LMWG. By introducing an additional carboxylate group into a known self-assembling motif, we aim to explore how molecular-level modifications enable targeted interactions with specific metal ions.^17^ This approach supports broader efforts to create responsive supramolecular materials with relevance to environmental remediation, resource recovery, and soft nanotechnology.^10,12,36^

## Results and discussion

**Fig. 1:**
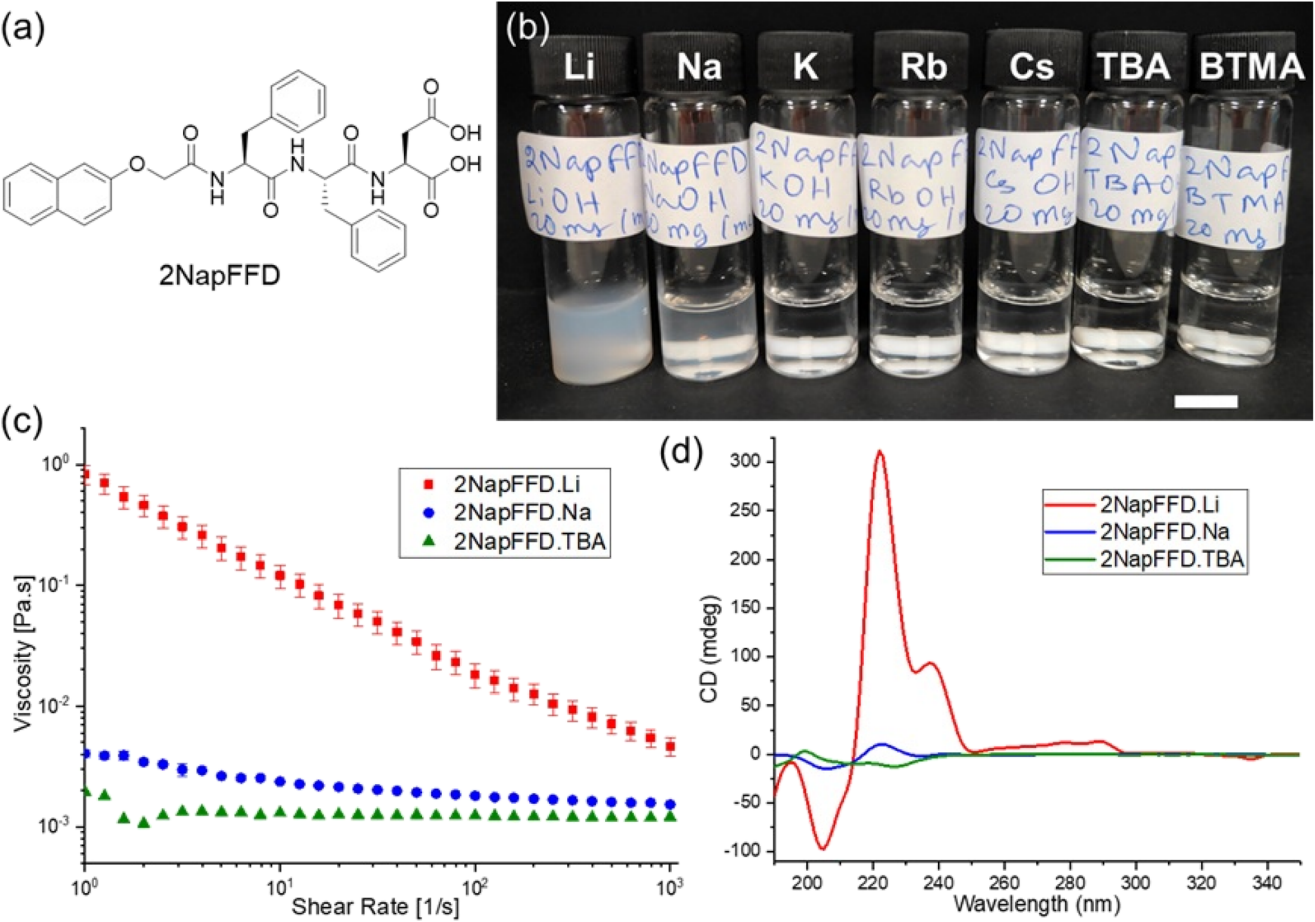
(a) Chemical structure of the tripeptide gelator 2NapFFD. (b) Photographs of 2NapFFD solutions (20 mg mL^−1^, pH 10.5) prepared using hydroxide bases with different counterions (Li^+^, Na^+^, K^+^, Rb^+^, Cs^+^, TBA^+^, and BTMA^+^). Scale bar: 1 cm. (c) Viscosity as a function of shear rate for 2NapFFD solutions prepared with LiOH, NaOH, and TBAOH (20 mg mL^−1^, pH 10.5, 25 °C). (d) CD spectra of 2NapFFD solutions prepared with LiOH, NaOH, and TBAOH (20 mg mL^−1^, pH 10.5), corresponding HT data are shown in Fig. S14.

We initially chose the tripeptide 2NapFFD (Fig. 1a) as the main gelator for this study because the self-assembly of the corresponding dipeptide 2NapFF has been widely investigated.^4,15^ Comparing the self-assembly of 2NapFF and 2NapFFD allows us to evaluate the effect of the additional carboxylic acid group and the increased hydrophilicity introduced by the aspartic acid residue. The tripeptide 2NapFFD was synthesised *via* standard peptide coupling methods, starting with the formation of a dipeptide from Boc-protected l-phenylalanine and l-phenylalanine methyl ester (Scheme S1).^39^ After ester hydrolysis, dimethyl l-aspartate was coupled, followed by Boc deprotection. The aromatic group was introduced at the N-terminus, and final ester hydrolysis afforded the title compound (full characterisation data for all compounds are shown in Fig. S1–S12). We first assessed self-assembly by dissolving 2NapFFD at high pH in the presence of a series of monovalent hydroxide bases (Fig. 1b). All solutions were adjusted to a concentration of 20 mg mL^−1^ and a pH of 10.5. Hydroxides of all Group 1 metals (Li^+^, Na^+^, K^+^, Rb^+^, Cs^+^) and two organic bases tetrabutylammonium (TBA^+^) and benzyltrimethylammonium (BTMA^+^) hydroxide were used to prepare 2NapFFD solutions. Among the seven cations tested, only 2NapFFD·Li^+^ solution exhibited a distinctly viscous character (Fig. 1b), whereas the other systems remained fluid. This observation indicates specific recognition of Li^+^ by the tripeptide. In contrast, the TBA^+^ and BTMA^+^ samples showed the lowest apparent viscosity and behaved as free-flowing solutions. To investigate cation-dependent self-assembly in more detail, we focused on Li^+^, Na^+^, and TBA^+^. Li^+^ gave a distinctly viscous solution, Na^+^ is the closest matching ion, and by contrast TBA^+^ is a bulky, non-coordinating organic cation. Viscosity measurements confirmed that LiOH-based solutions were at least an order of magnitude more viscous than those containing NaOH or TBAOH (Fig. 1c). In addition, both Li^+^ and Na^+^ solutions displayed shear-thinning behaviour characteristic of micellar assemblies, while the TBA^+^ solution had a viscosity comparable to water. We performed cross-polarised optical microscopy on the 2NapFFD solutions, and only the Li^+^ sample showed clear birefringent textures, consistent with the presence of ordered anisotropic assemblies, whereas the Na^+^ and TBA^+^ samples were featureless and showed no birefringence (Fig. S13).

The selectivity for Li^+^ may arise due to the presence of two closely spaced carboxylates in the C-terminal Asp, creating a high charge-density O-donor environment at high pH. Li^+^, being the smallest and hardest alkali metal ion, is expected to strongly associate with the carboxylate-rich 2NapFFD assemblies through compact ion pairing and charge compensation, thereby reducing electrostatic repulsion and enabling tighter packing and micellar growth.^16,32,40^ Li-aspartate coordination is well documented in solid-state crystal structures of lithium aspartate salts,^34^ but how such interactions translate to dynamic micellar or gel states remains underexplored.^41,42^ In contrast, the larger Na^+^ ion is expected to provide weaker charge compensation and less effective bridging at these sites, leading to reduced aggregation. For TBA^+^, the bulky and non-coordinating nature of the cation is unlikely to compensate the anionic carboxylate sites, so the local charge density remains high and tight packing is unfavoured, limiting micellar growth.

The self-assembly of 2NapFFD in the presence of Li, Na, and TBA was further examined using circular dichroism (CD) spectroscopy. Solutions were prepared at 20 mg mL^−1^ and pH 10.5 and measured in a 0.01 mm path length cuvette. The 2NapFFD·Li sample exhibited strong CD signals (Fig. 1d and S14), while 2NapFFD·Na and 2NapFFD·TBA were close to baseline. The 2NapFFD·Li spectrum shows a bisignate feature between 200–230 nm, with an intense positive band at 221 nm and a negative band at 205 nm. For naphthalene-capped peptides, this wavelength region is dominated by exciton coupling of the naphthalene chromophore (isolated naphthalene absorbs strongly from 200–230 nm with a maximum near 225 nm), and exciton coupling gives rise to paired positive and negative Cotton effects.^43,44^ Any peptide backbone contributions in this region (π → π* near 195 nm and the weaker n → π* near 220 nm) are expected to be much smaller and can be masked by the aromatic exciton-couplet, so we do not assign the 221 nm band to secondary structures such as β-sheet based on CD alone. A weaker feature at 237 nm is close to the onset of the naphthalene short-axis transition region and likely reflects additional ordering of the aromatic chromophores within the aggregates. In contrast, 2NapFFD·Na and 2NapFFD·TBA displayed only weak signals across 200–240 nm, indicating a much lower degree of chiral aromatic coupling and therefore less ordered aggregation under these conditions.

**Fig. 2:**
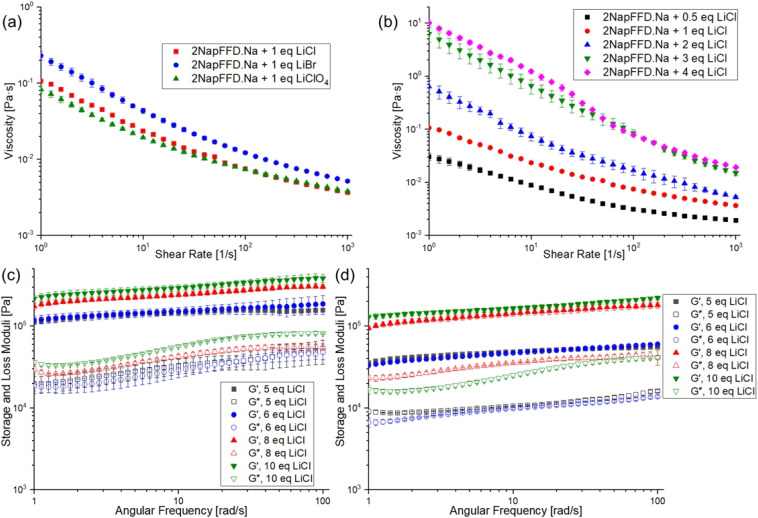
(a) Viscosity of 2NapFFD·Na (20 mg mL^−1^, pH 10.5) after addition of 1 equiv. of LiCl, LiBr, or LiClO_4_. (b) Viscosity of 2NapFFD·Na with increasing amount of added LiCl (0.5–4 equiv.). (c) Oscillatory frequency sweeps of gels formed from 2NapFFD·Na at higher amounts of LiCl (5–10 equiv.). (d) Oscillatory frequency sweeps of gels formed from 2NapFFD·TBA at higher LiCl loadings (5–10 equiv.).

To evaluate cation selectivity, we tested additional lithium salts LiBr and LiClO_4_ (Fig. 2a) and other Group 1 chloride salts (NaCl and KCl) as control experiments. The viscosity profiles of 2NapFFD·Na were similar upon adding LiCl, LiBr, or LiClO_4_, indicating that the anion has minimal influence on self-assembly and that Li^+^ governs the aggregation behaviour. In contrast, adding up to 5 equivalents of NaCl or KCl to 2NapFFD·Na produced no observable change in viscosity, consistent with Li^+^-specific triggering under these conditions (Fig. S15).

To further investigate selective Li-binding, we introduced different amounts of LiCl to the 2NapFFD·Na solution (Fig. S16). Adding 2 molar equivalents of LiCl to 2NapFFD·Na increased its viscosity similar to 2NapFFD·Li (Fig. 2b), indicating that the assembly reorganises in presence of Li^+^. We added excess LiCl to 2NapFFD·Na solution to examine how Li^+^ concentration influences the aggregated state. LiCl was added either as a solid or as an aqueous solution, and in both cases the viscosity increased with LiCl concentration, with the aqueous addition requiring slightly higher equivalents due to dilution. To avoid dilution and keep the 2NapFFD concentration constant, subsequent experiments were performed by adding solid salts, and all molar equivalents reported are relative to 2NapFFD unless stated otherwise. Samples containing ≥4 equivalents of LiCl were stable under vial inversion. However, frequency sweep experiments showed that the 4-equivalent sample remained a highly viscous solution rather than a true gel, and gel behaviour emerged at ≥5 equivalents of LiCl. We measured viscosity after adding 0.5–4 equivalents of LiCl (Fig. 2b) and performed frequency sweep rheology with 5–10 equivalents, which showed gelation was achieved for both 2NapFFD·Na and 2NapFFD·TBA at ≥5 equivalents of added LiCl (Fig. 2c and d).

**Fig. 3:**
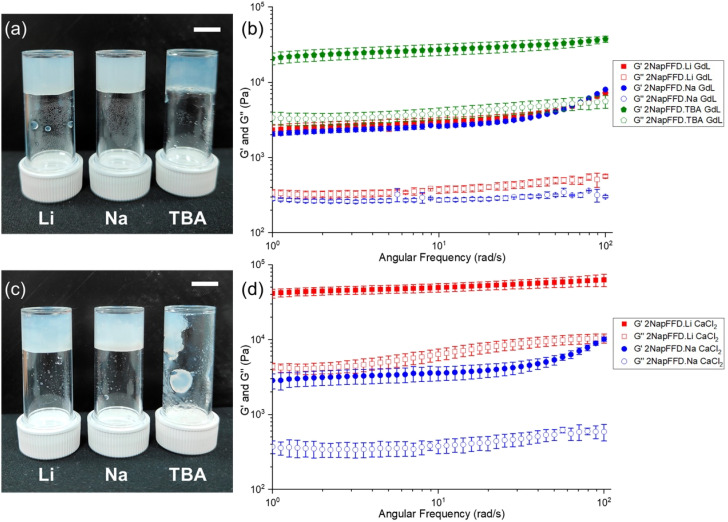
(a) Photographs of gels formed after mixing GdL and 2NapFFD solutions prepared with Li^+^, Na^+^, and TBA^+^. (b) Oscillatory frequency sweep of the corresponding GdL-triggered gels. (c) Photographs after adding CaCl_2_ to 2NapFFD solutions prepared with Li^+^, Na^+^, and TBA^+^ counterions. (d) Oscillatory frequency sweep of the CaCl_2_-triggered gels formed from the Li^+^ and Na^+^ solutions. Scale bars: 1 cm.

We compared the response of 2NapFFD to two common gelation triggers, GdL and CaCl_2_. On adding GdL, which results in a decrease in the pH to ∼3.8, all three systems (Li, Na, and TBA salts) formed gels.^45,46^ Frequency sweep rheology showed *G*′ greater than *G*″ across the measured range (Fig. 3a). The Li and Na gels had similar stiffness, whereas the gel formed from the TBA salt was substantially stiffer. The slow hydrolysis of GdL and resulting gradual pH decrease gives all systems enough time to reorganise and reassemble into a gel network, explaining why even the TBA salt ultimately forms a gel. In contrast, on adding 3 molar equivalents of CaCl_2_ solution, which is expected to cross-link micellar structures,^47^ only the Li- and Na-samples formed gels, and 2NapFFD·Li + CaCl_2_ gave a stiffer gel than the corresponding Na sample (Fig. 3b). This is consistent with Ca^2+^ rapidly cross-linking pre-existing micellar assemblies through carboxylate binding; the Li-system contains a higher population of micellar structures, while the Na-system likely contains fewer or shorter assemblies and the TBA-system even fewer, so Ca^2+^ cannot generate a stable network with the TBA salt. We also recorded ATR-FTIR spectra in D_2_O for gels formed using the different triggers (Fig. S17). The LiCl- and CaCl_2_-triggered gels showed broadly similar spectra, whereas the GdL-triggered gel showed a distinct profile, consistent with a protonated state. The LiCl- and CaCl_2_-triggered gels displayed an intense band at ∼1575 cm^−1^ (Fig. S18), which can be assigned primarily to the asymmetric stretching vibration of deprotonated carboxylate groups;^48^ this feature was not observed in the GdL gel.

**Fig. 4:**
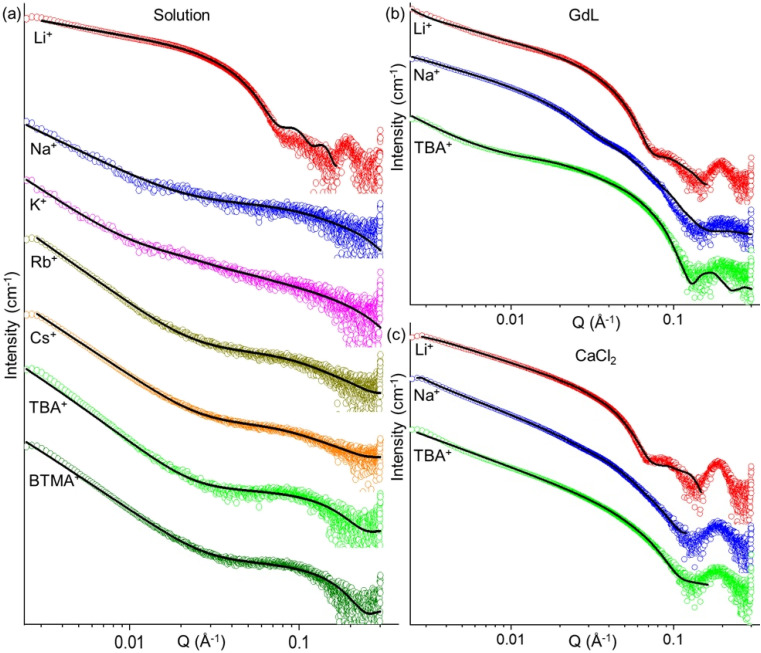
SAXS profiles of (a) 2NapFFD solutions prepared with different counterions (Li^+^, Na^+^, K^+^, Rb^+^, Cs^+^, TBA^+^, and BTMA^+^). (b) 2NapFFD (Li^+^, Na^+^, and TBA^+^ salts) after GdL addition. (c) 2NapFFD (Li^+^, Na^+^, and TBA^+^ salts) after CaCl_2_ addition. In all cases, the data are shown as empty circles and the fits as black lines. The data are shown as vertically stacked panels with individual *Y*-axis limits chosen to provide comparable visual heights (apparent scaling factors: (a) Li: 1, Na: 1.4, K: 20, Rb: 6.7, Cs: 0.6, TBA: 2, BTMA: 1; (b) Li: 1, Na: 2.4, TBA: 2.4; (c) Li: 1, Na: 2.8, TBA: 1.4); error bars are omitted for clarity. Corresponding fitting parameters are provided in Tables S1–S4.

We analysed the self-assembly of 2NapFFD by small-angle X-ray scattering (SAXS). SAXS data showed that solutions prepared with cations other than Li^+^ were well described by a power law plus cylinder model (Fig. 4a). The lengths of the fitted cylinders were very short within the accessible fitting range, typically 30–50 Å (Table S1), consistent with the low viscosity of these solutions. In contrast, the Li^+^ sample required a combination of cylinder and hollow cylinder model (Table S2), consistent with formation of tubular or core–shell like micelles. The Li^+^ dataset also showed a Bragg peak at the high *q* region, and this was excluded from fitting. The fitted objects were substantially longer than the non-Li^+^ cylinders, consistent with extended micellar assemblies. We also analysed the SAXS patterns after adding GdL to 2NapFFD solutions prepared with Li^+^, Na^+^, and TBA^+^ counterions (Fig. 4b and Table S3). The Li^+^ and Na^+^ samples were best described by elliptical cylinder type models, whereas the TBA^+^ sample was described by a cylinder with a power law contribution, suggesting that Li^+^ and Na^+^ shift the local packing towards a more anisotropic cross-section. The Na^+^ and TBA^+^ SAXS datasets obtained after adding CaCl_2_ (Fig. 4c and Table S4) could be fitted using flexible cylindrical models, but the Li^+^ sample required combination of two types of cylinders, consistent with the complex pre-assembled micellar state.

**Fig. 5:**
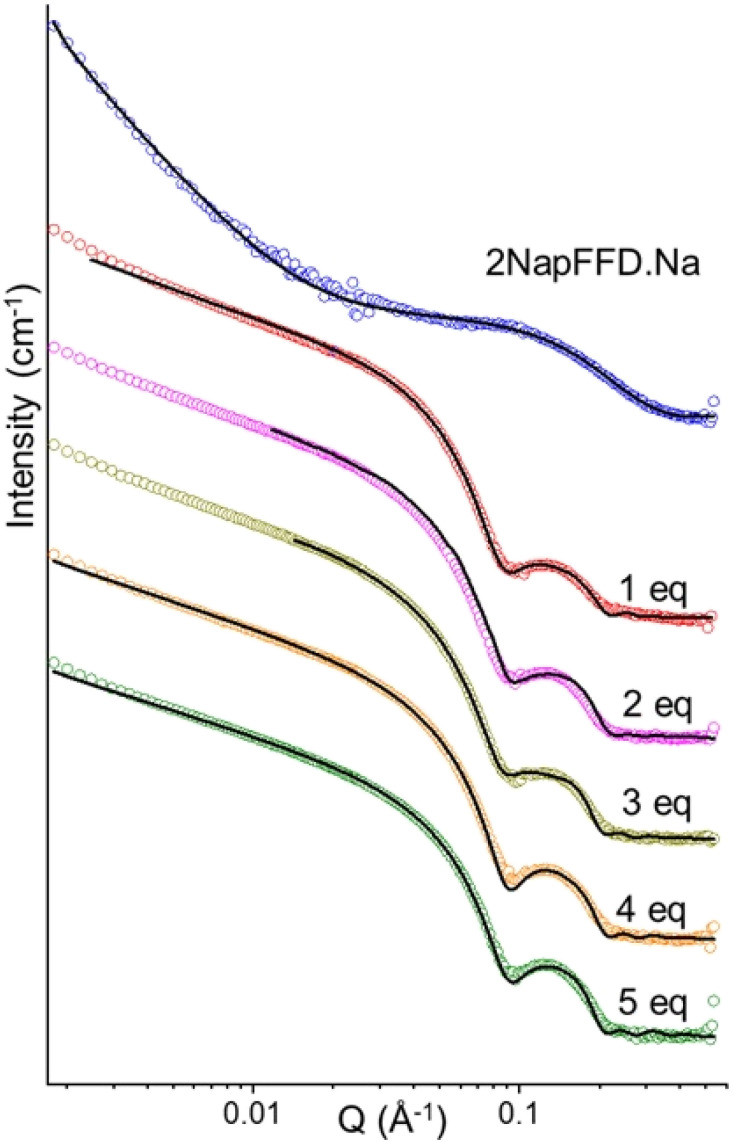
SANS profiles of 2NapFFD·Na (20 mg mL^−1^, pD 10.9, D_2_O) after addition of LiCl (1–5 equivalents relative to 2NapFFD), showing the evolution of the scattering pattern with increasing Li^+^ concentration. In all cases, the data are shown as empty circles and the fits as black lines. The data are shown as vertically stacked panels with individual *Y*-axis limits chosen to provide comparable visual heights (apparent scaling factors: Na: 1, 1 eq. Li: 3, 2 eq. Li: 6, 3 eq. Li: 5.5, 4 eq. Li: 8.7, 5 eq. Li: 17); error bars are omitted for clarity.

Next, to analyse the effect of excess Li^+^ on 2NapFFD, we added 1 to 5 molar equivalents of solid LiCl to a 2NapFFD·Na solution. It was difficult to prepare homogeneous mixtures in capillaries for SAXS, so we performed small-angle neutron scattering (SANS) in a Hellma cuvette using D_2_O instead of H_2_O for contrast. The 2NapFFD·Na solution without added Li^+^ was again best described by a cylinder plus power law model (Table S5), showing that the change in solvent has had no effect. After adding LiCl, the scattering converted to the Li-like profile and was best fitted to a cylinder plus hollow cylinder model (failed fits in other models are shown in Fig. S19), consistent with the SAXS data (Fig. 5). With increasing Li^+^ concentration, the fitted cylinder length increased, and at 2 equivalents of LiCl, the length exceeded the accessible fitting range, indicating the growth of long micelles and the formation of gels at higher Li^+^ concentrations. Replacing LiCl with LiBr gave similar scattering profiles and fitted parameters (Fig. S20 and Table S6), while adding NaCl or KCl (up to 5 equivalents) to 2NapFFD·Na produced no significant change, supporting Li^+^-specific assembly (Fig. S21 and Table S7).

**Fig. 6:**
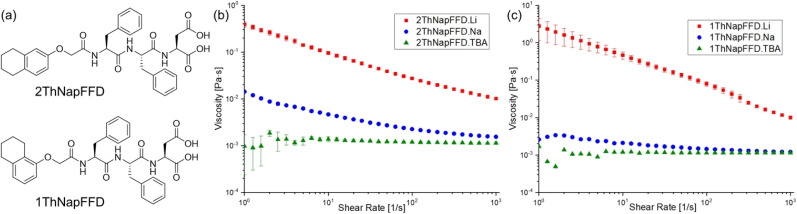
(a) Chemical structures of the tripeptide gelators 2ThNapFFD and 1ThNapFFD. (b) Viscosity of 2ThNapFFD solutions prepared with LiOH, NaOH, and TBAOH (20 mg mL^−1^, pH 10.5, 25 °C). (c) Viscosity of 1ThNapFFD solutions prepared with LiOH, NaOH, and TBAOH (20 mg mL^−1^, pH 10.5, 25 °C).

To investigate the contribution of the FFD tripeptide moiety to Li^+^ selectivity, we synthesised two analogues of 2NapFFD: 2ThNapFFD and 1ThNapFFD (Th = tetrahydro; see Fig. 6a and Scheme S2). Solutions of both tripeptides were prepared with all seven cations at 20 mg mL^−1^ and pH 10.5. As observed for 2NapFFD, the Li^+^-based solutions of 2ThNapFFD and 1ThNapFFD formed viscous micellar solutions, whereas solutions with other cations remained clear and fluid. We performed viscosity measurements for the Li^+^, Na^+^, and TBA^+^ salts of both 2ThNapFFD and 1ThNapFFD. In each case, the Li^+^-based solutions were more viscous than the corresponding Na^+^ and TBA^+^ samples (Fig. 6b and c), while the Na^+^ solutions showed only weak shear-thinning and the TBA^+^ solutions remained close to water-like. We performed cross-polarised microscopy and observed clear birefringent domains for 1ThNapFFD·Li, whereas 2ThNapFFD·Li remained non-birefringent under the same conditions (Fig. S22 and S23).

**Fig. 7:**
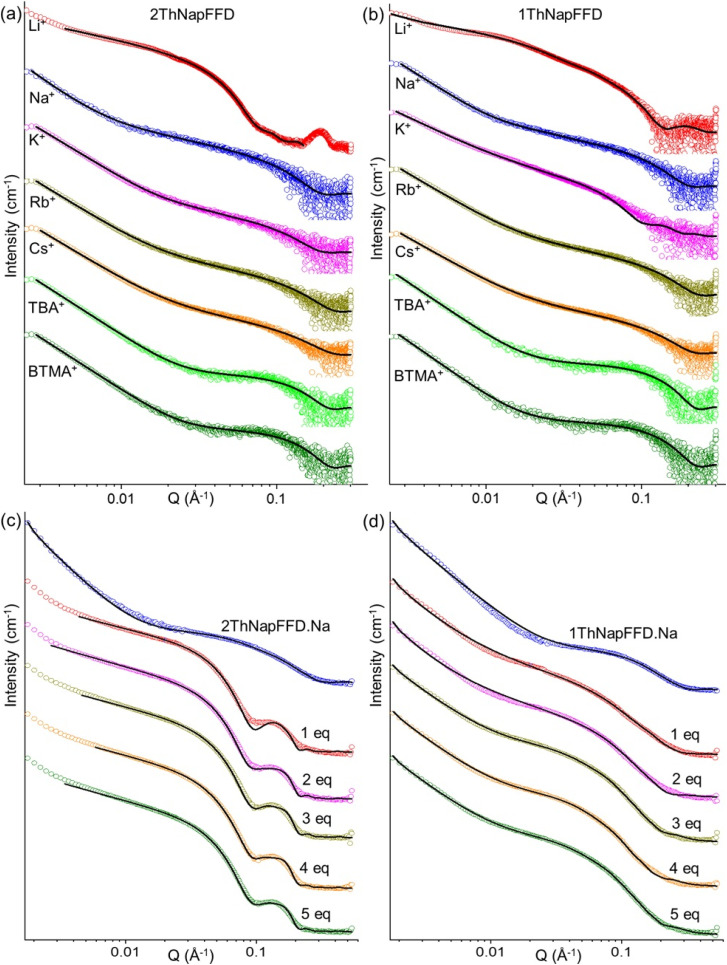
SAXS profiles of (a) 2ThNapFFD and (b) 1ThNapFFD solutions prepared with different counterions (Li^+^, Na^+^, K^+^, Rb^+^, Cs^+^, TBA^+^, and BTMA^+^). SANS profiles of (c) 2ThNapFFD·Na and (d) 1ThNapFFD·Na upon addition of LiCl (1–5 equivalents relative to the peptide), showing the evolution of the scattering pattern with increasing Li^+^ concentration. In all cases, the data are shown as empty circles and the fits as black lines. The data are shown as vertically stacked panels with individual *Y*-axis limits chosen to provide comparable visual heights (apparent scaling factors: (a) Li: 1, Na: 0.5, K: 0.08, Rb: 0.12, Cs: 0.11, TBA: 0.1, BTMA: 0.1; (b) Li: 1, Na: 3, K: 0.6, Rb: 1.08, Cs: 0.67, TBA: 5.6, BTMA: 8.4; (c) Na: 1, 1 eq. Li: 1.2, 2 eq. Li: 1.1, 3 eq. Li: 0.8, 4 eq. Li: 1.07, 5 eq. Li: 1.2; (d) Na: 1, 1 eq. Li: 0.83, 2 eq. Li: 0.83, 3 eq. Li: 0.91, 4 eq. Li: 1, 5 eq. Li: 1); error bars are omitted for clarity.

We also analysed the scattering patterns of 2ThNapFFD and 1ThNapFFD to assess whether the Li-triggered behaviour is general across different N-terminal aromatics. SAXS data showed that solutions of both tripeptides prepared with bases other than LiOH were again well described by a power law plus cylinder model (Fig. 7a and b), and the fitted micelle lengths and diameters were comparable to the corresponding 2NapFFD samples (Tables S8–S11), indicating similar micellar structures. For the Li^+^ salts, 2ThNapFFD·Li required a cylinder plus hollow cylinder model and showed a distinct high-*q* peak similar to 2NapFFD·Li, which was excluded from fitting. In contrast, 1ThNapFFD·Li was best fitted in a power law plus elliptical cylinder model, suggesting a different cross-sectional geometry in the Li-induced assemblies. Consistent with the SAXS trends, SANS measurements on samples prepared by adding LiCl to the Na^+^ salts of 2ThNapFFD and 1ThNapFFD showed the same evolution, with the scattering shifting to Li-like patterns and the fitted cylinder lengths increasing as the Li^+^ concentration increased (Fig. 7c, d, Tables S12 and S13). Together, these results indicate that Li^+^ selectivity is an intrinsic feature of the FFD tripeptide, while the N-terminal aromatic group modulates the packing geometry and the robustness of the resulting micellar structures.

To evaluate the importance of the tripeptide FFD moiety in cation-induced self-assembly, we designed a dipeptide analogue, 2NapFD, containing one phenylalanine residue and an aspartic acid (Fig. S24a). The 2NapFD solutions prepared with LiOH, NaOH, and TBAOH exhibited viscosities comparable to water (Fig. S24b) and no birefringence under polarised light (Fig. S25), indicating no sign of viscous aggregation on the macroscopic scale. This behaviour may reflect the decreased hydrophobicity associated with removing one phenylalanine residue, which likely disrupts the amphiphilic balance required for the formation of extended micelles. We collected SAXS data for 2NapFD with Li^+^, Na^+^, and TBA^+^ counterions (Fig. S26), and in all three cases the profiles were well described by a power law plus cylinder model (Table S14). Although the fitted cylinder dimensions varied between salts, the objects remained short and the fits did not indicate growth of long micelles as observed for the tripeptides. Overall, these results suggest that 2NapFD forms only weak, short aggregates, and that Li-driven micellar growth requires the full FFD tripeptide framework.

**Fig. 8:**
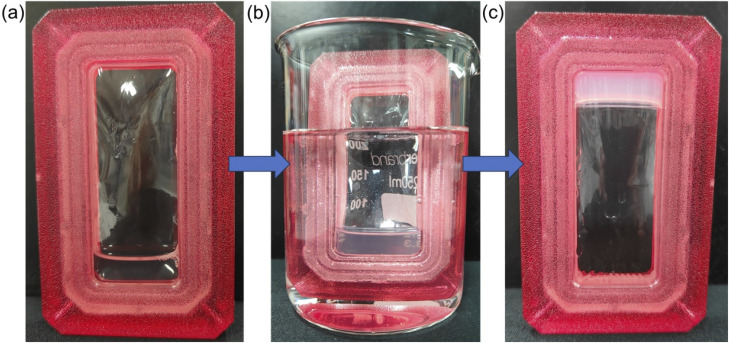
Dialysis-based Li^+^ capture and gelation of 2NapFFD. (a) 2NapFFD·Na solution loaded in a dialysis cassette before dialysis. (b) Dialysis cassette immersed in an external LiCl solution (70 mM). (c) After overnight dialysis, the solution inside the cassette forms a translucent gel; the cassette is inverted to show the formation of gel.

Leveraging the Li^+^ selectivity of 2NapFFD, we investigated whether the peptide solution could capture Li^+^ from an external reservoir. For this, we dialysed a 2NapFFD·Na solution against LiCl. A 2 mL aliquot of 2NapFFD·Na (20 mg mL^−1^, pH 10.5) was placed in a 12 mL dialysis cassette and immersed in a 250 mL beaker. The Na^+^ concentration in the peptide solution was estimated from the NaOH used for dissolution and pH adjustment, giving a final Na^+^ concentration of approximately 70 mM. Accordingly, the peptide solution was dialysed against 20 mL of 70 mM LiCl to provide a large external reservoir of Li^+^ and maintain favourable diffusion conditions (Fig. 8). After overnight dialysis, the 2NapFFD·Na solution had transformed into a translucent gel, indicating that Li^+^ diffused across the membrane and induced self-assembly. In a control experiment, dialysis of 2NapFFD·Na against 70 mM NaCl resulted in no change in the solution state. These results support the finding that Li^+^ selectively triggers gelation by diffusing into the 2NapFFD·Na solution and promoting self-assembly.

Finally, we quantified Li^+^ uptake by 2NapFFD using inductively coupled plasma optical emission spectroscopy (ICP-OES) by tracking Li^+^ and Na^+^ concentrations in the external dialysate before and after dialysis. Three dialysate solutions were prepared: 70 mM LiCl, 70 mM NaCl, and a 1 : 1 mixture (35 mM each) of LiCl/NaCl (Fig. S27). After dialysis, Li^+^ and Na^+^ concentrations in the external dialysate were measured, and the changes from the starting values were used to quantify net Li^+^ uptake (and Na^+^ release) by the peptide solution. Dialysis of 2NapFFD·Na against NaCl resulted in a small decrease in Na^+^ concentration, from 69.0 mM to 66.6 ± 1.38 mM (Table 1), which may reflect weak association of Na^+^ with the peptide and redistribution across the membrane to maintain equilibrium. In contrast, dialysis against LiCl produced a marked decrease in Li^+^ concentration, from 68.4 mM to 50.4 ± 0.55 mM, indicating substantial Li^+^ uptake and consistent with Li^+^-triggered gelation. The data show that 2 mL of 20 mg per mL 2NapFFD·Na (40 mg, 65 µmol, eq. to 130 µmol of COOH^−^) was able to capture ∼360 µmol of Li^+^ (∼2.75 Li^+^ per COOH^−^). This is in good agreement with the gelation studies, which showed 2NapFFD·Na forms a gel at over 5 eq. of LiCl (2.5 Li^+^ per COOH^−^). When 2NapFFD·Na was dialysed against the 1 : 1 LiCl/NaCl mixture, the concentration of Li^+^ decreased from 34.5 mM to 27.2 ± 0.07 mM while Na^+^ increased from 34.4 mM to 40.7 ± 2.2 mM, suggesting Li^+^ uptake coupled to Na^+^ release. The solution inside the cassette became viscous but did not form a gel, presumably because the concentration of the Li^+^ in the reservoir was much lower than the Na^+^ concentration in the original solution. Overall, these results demonstrate selective Li^+^ uptake by 2NapFFD over Na^+^, highlighting potential for lithium capture and separation.

**Table 1:** Quantification of Li^+^ uptake by 2NapFFD·Na

Ions	Concentration before dialysis (mM)	Concentration after dialysis^a^ (mM)	Li^+^ uptake by 2 mL (40 mg, 65 µmol) 2NapFFD (µmol)
Li^+^	68.4	50.4 (±0.55)	360.0 (±11)
Na^+^	69.0	66.6 (±1.38)	—
Li^+^ + Na^+^	Li^+^: 34.5	Li^+^: 27.2 (±0.07)	146.0 (±1.4)
	Na^+^: 34.4	Na^+^: 40.7(±2.2)	

a Dialysing 2 mL DI water would lead the final Li^+^ ion concentration to 62.1 mM and 31.4 mM in rows 1 and 3, respectively.

## Experimental

### Materials and methods

Reagents and solvents were obtained from commercial suppliers (Sigma-Aldrich or Fluorochem Ltd) and used without further purification. Deionised water was used throughout. The peptide conjugates and ThNapOCH_2_COOH were prepared following previously reported procedures.^39,47^ Full synthetic protocols are given below.

### Synthesis of the peptides

To a stirred solution of the N-terminal capping acid (2NapOCH_2_CO_2_H or ThNapOCH_2_CO_2_H, 5.0 mmol, 1.0 equiv.) in CHCl_3_ (70 mL) at 0 to 5 °C were added *N*-methylmorpholine (6.0 mmol, 1.2 equiv.), followed by isobutyl chloroformate (6.0 mmol, 1.2 equiv.). The reaction mixture was stirred for 1 h in an ice bath to generate the mixed anhydride. Separately, a solution of the dimethyl ester protected peptide (FFD(OMe)_2_ or FD(OMe)_2_) was prepared by stirring the TFA salt of the peptide (5.0 mmol, 1.0 equiv.) with *N*-methylmorpholine (7.0 mmol, 1.4 equiv.) in CHCl_3_ (50 mL) in an ice bath. This peptide solution was added to the mixed anhydride solution, and the reaction was allowed to warm to ambient temperature and stirred overnight. The mixture was diluted with CHCl_3_ and washed sequentially with 1 M HCl, water, and brine. The organic phase was dried over MgSO_4_, filtered, and concentrated under reduced pressure to afford the crude coupled product, which was used in the next step without further purification.

The crude ester (4.0 mmol, 1.0 equiv.) was dissolved in THF (25 mL) and treated with an aqueous LiOH solution (1.0 M, 25 mL, 6.2 equiv.). The mixture (initially biphasic or cloudy) was stirred at ambient temperature overnight and typically became homogeneous as the hydrolysis proceeded. The reaction mixture was poured into 0.5 M HCl (250 mL) and stirred for 60 to 90 min to ensure complete protonation and precipitation. The resulting white solid was collected by filtration, washed thoroughly with water and once with MeCN, and dried in a vacuum oven to remove residual solvent.

### Characterisation of the peptides

#### 2NapFFD

Yield: 2.22 g, 3.63 mmol (90.7%). ^1^H NMR (400 MHz, DMSO) *δ* 12.63 (s, 2H), 8.44 (d, *J* = 7.9 Hz, 1H), 8.31 (d, *J* = 8.4 Hz, 1H), 8.05 (d, *J* = 8.4 Hz, 1H), 7.88–7.79 (m, 2H), 7.74 (d, *J* = 8.2 Hz, 1H), 7.47 (ddd, *J* = 8.2, 6.7, 1.3 Hz, 1H), 7.37 (ddd, *J* = 8.2, 6.7, 1.2 Hz, 1H), 7.30–7.07 (m, 12H), 4.60 (ddd, *J* = 14.8, 8.2, 3.1 Hz, 3H), 4.53 (d, *J* = 1.9 Hz, 2H), 3.11–2.95 (m, 2H), 2.87–2.58 (m, 4H). ^13^C NMR (101 MHz, DMSO) *δ* 172.22, 171.65, 170.82, 170.49, 167.19, 155.46, 137.53, 137.48, 134.03, 129.37, 129.26, 129.24, 128.77, 128.00, 127.95, 127.50, 126.81, 126.43, 126.25, 126.19, 123.87, 118.42, 107.40, 66.73, 53.57, 53.32, 48.69, 37.65, 37.41, 36.01. HRMS [M–H]^−^ calculated for [C_34_H_32_N_3_O_8_]^−^: 610.2195, found 610.2205.

#### 2ThNapFFD

Yield: 2.08 g, 3.38 mmol (84.6%). ^1^H NMR (400 MHz, DMSO) *δ* 12.61 (s, 2H), 8.43 (d, *J* = 7.9 Hz, 1H), 8.28 (d, *J* = 8.4 Hz, 1H), 7.86 (d, *J* = 8.4 Hz, 1H), 7.30–7.08 (m, 10H), 6.91 (d, *J* = 8.2 Hz, 1H), 6.61–6.49 (m, 2H), 4.58 (ddt, *J* = 14.2, 10.3, 5.4 Hz, 3H), 4.38–4.25 (m, 2H), 3.12–2.89 (m, 2H), 2.86–2.68 (m, 3H), 2.67–2.56 (m, 5H), 1.69 (m, 4H). ^13^C NMR (101 MHz, DMSO) *δ* 172.21, 171.64, 170.82, 170.42, 167.45, 155.38, 137.60, 137.53, 137.41, 129.71, 129.27, 129.24, 128.00, 127.95, 126.26, 126.20, 114.46, 112.36, 66.73, 53.53, 53.15, 48.66, 37.66, 37.41, 35.96, 29.01, 27.97, 22.93, 22.64. HRMS [M–H]^−^ calculated for [C_34_H_36_N_3_O_8_]^−^: 614.2508, found 614.2501.

#### 1ThNapFFD

Yield: 1.75 g, 2.84 mmol (71.1%, the yield was lower due to higher solubility of the material in MeCN). ^1^H NMR (400 MHz, DMSO) *δ* 12.64 (s, 2H), 8.47 (d, *J* = 7.9 Hz, 1H), 8.35 (d, *J* = 8.6 Hz, 1H), 7.67 (d, *J* = 8.4 Hz, 1H), 7.30–7.08 (m, 10H), 6.95 (t, *J* = 7.9 Hz, 1H), 6.67 (d, *J* = 7.6 Hz, 1H), 6.49 (d, *J* = 8.1 Hz, 1H), 4.69–4.53 (m, 3H), 4.44–4.28 (m, 2H), 3.11–2.93 (m, 2H), 2.89–2.75 (m, 2H), 2.75–2.42 (m, 6H), 1.77–1.58 (m, 4H). ^13^C NMR (101 MHz, DMSO) *δ* 172.21, 171.64, 170.87, 170.21, 167.26, 155.11, 137.93, 137.55, 137.10, 129.34, 129.26, 127.99, 127.97, 126.25, 125.74, 125.10, 121.87, 108.49, 66.78, 53.50, 52.84, 48.69, 37.74, 37.56, 35.99, 28.99, 22.56, 22.32. HRMS [M–H]^−^ calculated for [C_34_H_36_N_3_O_8_]^−^: 614.2508, found 614.2529.

#### 2NapFD

Yield: 1.70 g, 3.66 mmol (91.6%). ^1^H NMR (400 MHz, DMSO) *δ* 12.63 (s, 2H), 8.55 (d, *J* = 7.9 Hz, 1H), 8.18 (d, *J* = 8.6 Hz, 1H), 7.88–7.80 (m, 2H), 7.73 (dd, *J* = 8.3, 1.2 Hz, 1H), 7.47 (ddd, *J* = 8.2, 6.8, 1.3 Hz, 1H), 7.36 (ddd, *J* = 8.1, 6.8, 1.2 Hz, 1H), 7.27–7.20 (m, 2H), 7.20–7.12 (m, 5H), 4.67 (td, *J* = 9.1, 4.1 Hz, 1H), 4.63–4.50 (m, 3H), 3.08 (dd, *J* = 13.8, 4.1 Hz, 1H), 2.88 (dd, *J* = 13.8, 9.6 Hz, 1H), 2.72 (dd, *J* = 16.6, 5.8 Hz, 1H), 2.60 (dd, *J* = 16.6, 6.9 Hz, 1H). ^13^C NMR (101 MHz, DMSO) *δ* 172.32, 171.75, 170.87, 167.44, 155.58, 137.55, 134.12, 129.50, 129.35, 128.86, 128.12, 127.61, 126.90, 126.57, 126.42, 124.01, 118.54, 107.44, 66.77, 53.35, 48.83, 37.61, 36.04. HRMS [M–H]^−^ calculated for [C_25_H_23_N_2_O_7_]^−^: 463.1511, found 463.1517.

### Preparation of the peptide solutions

The peptide solutions were prepared by stirring 200 mg of peptide in 10 mL of deionised water with 2.0 equiv. of the appropriate base overnight. The mixture was stirred continuously for approximately 18 h at ambient temperature (around 20 °C) using a 25 × 8 mm stirrer bar in a 50 mL centrifuge tube at 750 rpm. The pH of the resulting solution was then adjusted to 10.5 ± 0.05 using 1.0 M of the same base.

### Viscosity measurements

Viscosity was measured using an Anton Paar Physica MCR 101 rheometer fitted with a 50 mm, 1° cone plate (CP50) at 25 °C. The instrument set truncation gap was 0.101 mm. Approximately 1 mL of each sample was carefully dispensed directly onto the plate to minimize shear associated with pipetting. Measurements were performed in triplicate, and results are reported as mean values with standard deviations.

### Thermal annealing

To assess thermal effects on sample behaviour, 2 mL of peptide solution (20 mg mL^−1^, pH 10.5) was transferred to a 7 mL glass vial and heated at 70 °C for 1 h, followed by cooling to room temperature. Gelation was assessed by the vial inversion test.

### Circular dichroism (CD)

A drop of the tripeptide solutions was transferred using a Pasteur pipette to the spacer window of a demountable cuvette with a path length of 0.01 mm. The liquid was spread as uniformly as possible across the spacer, then the cuvette was closed with a flat quartz window and sealed. Any solution expressed at the edges was removed carefully before placing the cuvette in the instrument. CD spectra were collected on a Chirascan CD spectrometer (Applied Photophysics) at 25 °C from 190 to 350 nm, using a step size of 1 nm and a bandwidth of 1 nm. Spectra represent the average of three accumulations. DI water was used as the background and was subtracted from each of the solution spectra, and the data were smoothed in OriginPro 2018 using a Savitzky–Golay filter with a 10-point window.

### Microscopy

Microscopic images were acquired on a Leica DM750 microscope using a 5× objective. For high pH solutions, a drop of the peptide solution was placed on a glass slide. The microscope was first calibrated under white light and brightfield images were collected, after which polarised illumination was enabled and the analyser and polariser were set to 90° to obtain polarised light images. Scale bars were added using MATLAB vR2025b.

### Rheology

Oscillatory frequency sweep measurements were performed on an Anton Paar Physica MCR101 rheometer. A 2 mL tripeptide solution (20 mg mL^−1^, pH 10.5) was transferred to a 7 mL Sterilin vial, and an appropriate trigger (GdL, CaCl_2_, or LiCl) was added. The sample was mixed by gentle shaking for about 10 s and then left undisturbed for 1 d to reach equilibrium. The vial was mounted on the rheometer and measurements were collected using a rotating vane geometry with a 10 mm diameter and a 1.8 mm gap between the vane and the base of the vial. Frequency sweeps were carried out at 25 °C at a fixed strain of 0.05%. Measurements were repeated three times and the mean values were reported, with error bars representing the standard deviation.

### Attenuated total reflectance-Fourier transform infrared (ATR-FTIR) spectroscopy

Infrared spectra were recorded on an Agilent Cary 630 FTIR spectrometer equipped with an ATR attachment. A 20 mg per mL solution of 2NapFFD·Na was prepared in a NaOD/D_2_O mixture as described above, and the pD was adjusted to 10.9 (pD = pH + 0.4). Solid LiCl, CaCl_2_, or GdL was placed in a vial, and 1 mL of the 2NapFFD·Na solution in D_2_O was added. The samples were vortexed and left overnight at room temperature. Liquid samples were applied to the ATR crystal using a Pasteur pipette, while gel samples were transferred using a spatula. Spectra were acquired over the range of 4000 to 600 cm^−1^ at a resolution of 2 cm^−1^, with 64 scans averaged for both the D_2_O background and each sample.

### Small-angle X-ray scattering (SAXS)

SAXS measurements were carried out on the I22 beamline at Diamond Light Source. The beamline was operated at a fixed energy of 13 keV with a camera length of 3.6 m, providing a *q* range of 0.00246 to 0.301 Å^−1^. Samples were prepared as described above and introduced into capillaries with 1.56 mm ID using a syringe fitted with a 21G needle. For each sample, five 0.5 s exposures were collected and averaged. The 2D scattering images were reduced in DAWN Science (v2.40)^49^ to generate *I vs. q* profiles. The aqueous background was subtracted in SasView (v5.0.6), and all 1D scattering data were fitted using SasView.

### Small-angle neutron scattering (SANS)

For SANS experiments, peptide solutions were prepared in a NaOD/D_2_O medium as described above and the pD was adjusted to 10.9. The solutions were loaded into Hellma quartz cuvettes with a 2 mm path length. Where required, LiCl was added as a solid to the prepared solutions prior to measurement. Measurements were performed on the D11 instrument at the Institut Laue Langevin (ILL) using sample-to-detector distances of 2.0 m, 10.2 m, and 38.9 m together with a neutron wavelength of 5.24 Å to cover the high, intermediate, and low *q* ranges. Data were reduced by subtracting the electronic background, normalizing the 2D detector images, and subtracting scattering contributions from the empty cell and D_2_O. The reduced data were converted to 1D scattering profiles (intensity *vs. q*) and merged to give a *q* range of 0.00179 to 0.536 Å^−1^ using GRASP.^50^ The reduced data were then analysed in SasView (v5.0.6), fitting to the models described in the main text.

### Dialysis experiment

2NapFFD-Na solution was prepared at 20 mg mL^−1^ and pH 10.5 as described above. The amount of base required to adjust the pH was tracked to calculate the final Na concentration in the mixture, which was approximately 70 mM. A 2 mL aliquot of the 2NapFFD-Na solution was transferred into a 12 mL dialysis cassette and placed in a 250 mL beaker. The solution was dialysed against ∼150 mL of 70 mM LiCl or NaCl solution overnight. The cassette was removed, the exterior was rinsed and dried, and gel formation inside the cassette was assessed by the vial inversion test.

### Inductively coupled plasma optical emission spectroscopy (ICP-OES)

To quantify the amount of Li^+^/Na^+^ uptake by the 2NapFFD-Na solution by ICP-OES, it was necessary to reduce the volume of the dialysing solution. A custom 3D-printed holder (Fig. S27) was prepared with inner dimensions slightly larger than the outer dimensions of the dialysis cassette. Three dialysis cassettes containing 2NapFFD-Na solution were placed inside three separate holders, and 20 mL of 70 mM NaCl, 70 mM LiCl, or a 1 : 1 mixture of both (35 mM NaCl and 35 mM LiCl) was added to the respective setups. The entire setup was placed in a 1 L beaker and sealed to prevent evaporation. After dialysing for 18 h, 20 µL aliquots of the external dialysate were diluted into 9.98 mL of deionised water to give a 500-fold dilution. The stock solutions before dialysis were also diluted 500-fold, and a deionised water blank was measured. ICP-OES was carried out on an Agilent 5900 instrument at the UoG School of Chemistry. The Na^+^ contribution from the deionised water blank was subtracted from all measured Na^+^ concentrations; Li^+^ was not detected in the deionised water blank. Experiments were repeated three times and the mean and standard deviation were reported.

## Conclusions

This study establishes the tripeptide FFD as a modular building block for Li^+^-selective self-assembly in N-capped peptide LMWGs. Under basic conditions, 2NapFFD shows a strong counterion dependence. Li^+^ uniquely produces highly viscous, shear-thinning solutions that can be converted into gels by increasing Li^+^ concentration, whereas Na^+^ and bulky organic counterions give weakly structured, low-viscous solutions. Small-angle scattering supports this behaviour, showing that Li^+^ promotes substantially more extended micellar assemblies than non-Li^+^ systems. The Li^+^ response generalises across different aromatic caps such as in 2ThNapFFD and 1ThNapFFD, indicating that selectivity originates from the FFD core, while the aromatic cap further tunes packing and mesoscale order. In contrast, the dipeptide control 2NapFD shows short aggregates and forms a water-like solution due to reduced hydrophobicity, preventing the formation of extended micellar networks. The Li^+^ selectivity may arise from the carboxylate-rich, high charge-density environment introduced by Asp. At high pH, the Asp carboxylate increases local anionic character, which disfavours tight packing unless efficiently compensated. Li^+^, being the smallest and hardest alkali cation, can effectively cross-link the carboxylate-rich assemblies by forming compact contact ion pairs, reducing electrostatic repulsion and enabling tighter packing and micellar elongation. Dialysis and ICP-OES quantify the Li^+^ uptake from an external reservoir, showing that each carboxylate anion on average can uptake ∼2.75 eq. of Li^+^. Overall, this work provides a tunable supramolecular platform for selective Li^+^ recognition and triggered gelation, and it opens the scope to optimise supramolecular design to improve selectivity for lithium capture, sensing, and recycling applications.

## Author contributions

Conceptualization: D. G.; data curation: D. G.; formal analysis: D. G., D. J. A.; funding acquisition: D. J. A.; investigation: D. G., D. J. A.; methodology: D. G.; project administration: D. J. A.; resources: D. J. A.; software: R. S., A. J. S.; supervision: D. J. A.; validation: D. G., R. S., D. J. A.; visualization: D. G., D. J. A.; writing – original draft: D. G.; writing – review & editing: D. G., R. S., D. J. A.

## Conflicts of interest

There are no conflicts to declare.

## Supplementary Material

SC-OLF-D6SC01183G-s001

## Data Availability

The data supporting this article have been included as part of the supplementary information (SI). Supplementary information: synthetic schemes; NMR spectra; additional viscosity data; microscopy and CD HT data; SAXS/SANS profiles with SasView fits and fitting parameters; dialysis and ICP-OES setup images. See DOI: https://doi.org/10.1039/d6sc01183g.
