# Supplementary material for: Lithium-selective supramolecular assembly and capture by tripeptide gelators
Source: Chem Sci. 2026 Apr 13;17(21):10456–68. doi: 10.1039/d6sc01183g (PMC13093460; doi:10.1039/d6sc01183g)
Supplement: SC-017-D6SC01183G-s001 [file SC-017-D6SC01183G-s001.pdf]

Supplementary Information (SI) for Chemical Science.
This journal is © The Royal Society of Chemistry 2026

# Supplementary information

# Lithium-Selective Supramolecular Assembly and Capture by Tripeptide Gelators

*Dipankar Ghosh,* [a] *Ralf Schweins,* [b] *Andrew J. Smith,* [c] *and Dave J. Adams** [a].

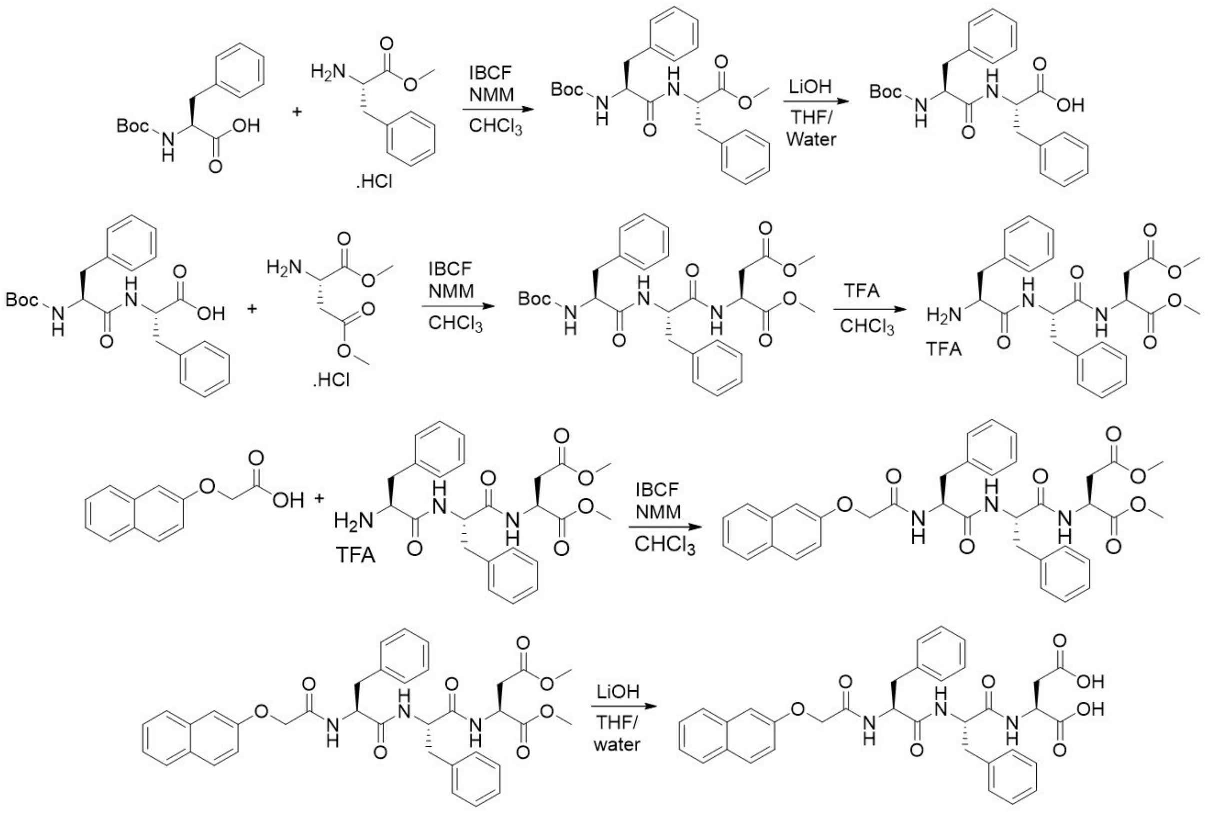


**Scheme S1:** Full synthetic route of the tripeptide gelator 2NapFFD.

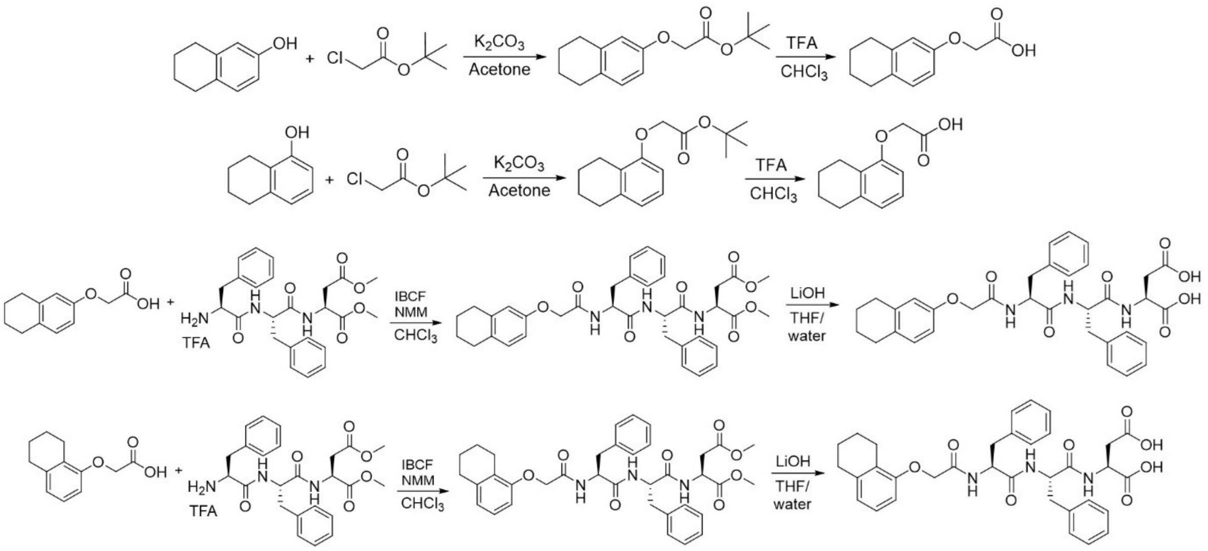

**Scheme S2:** Full synthetic route of the tripeptide gelators 2ThNapFFD and 1ThNapFFD.

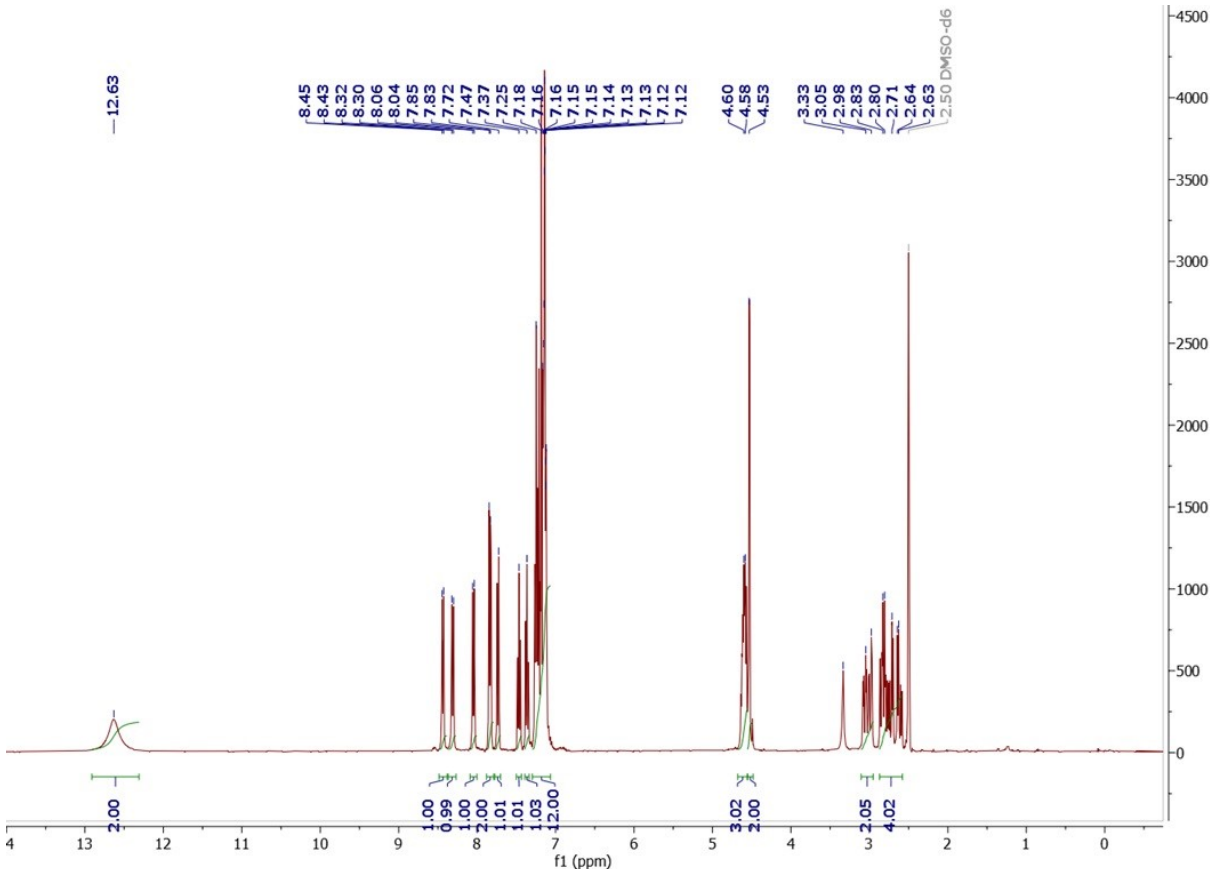

**Figure S1:** $^{1}$H-NMR of *2NapFFD.*

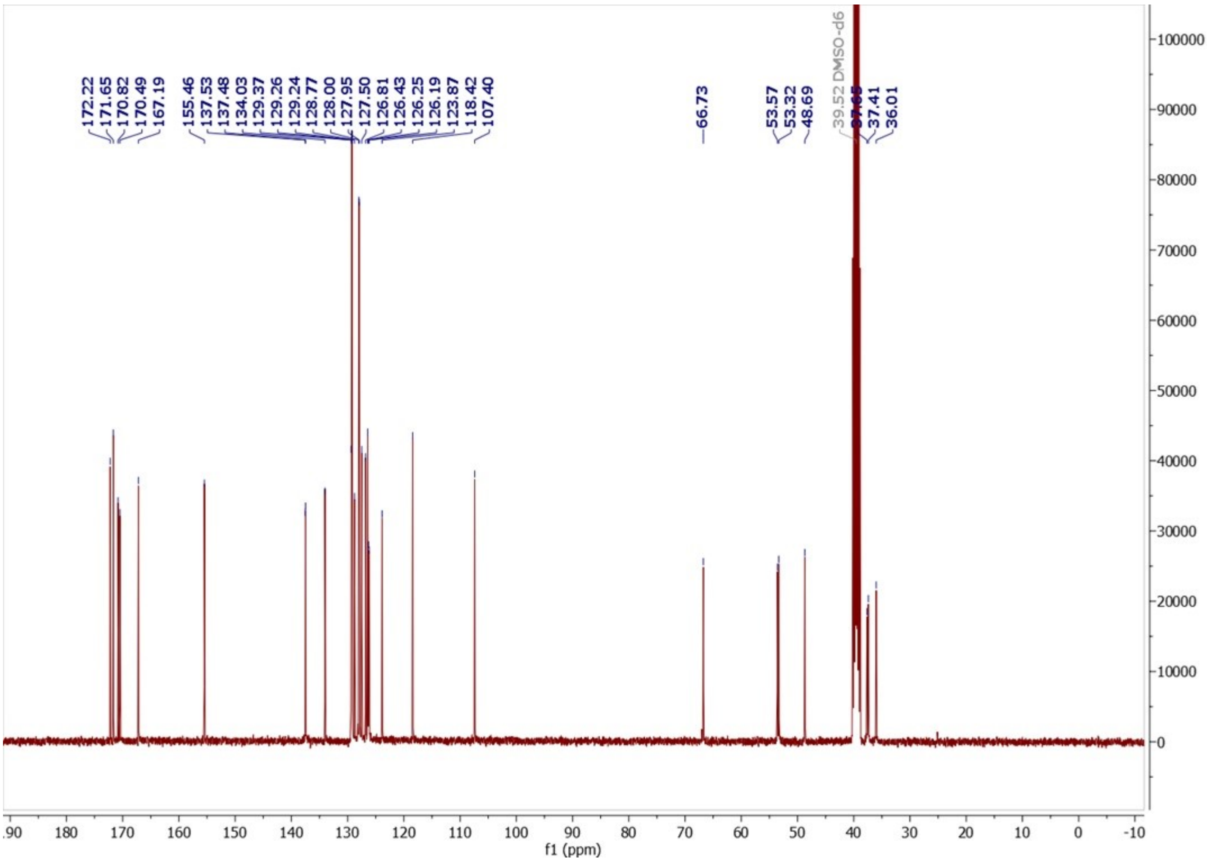


**Figure S2:** $^{13}$C-NMR of *2NapFFD.*

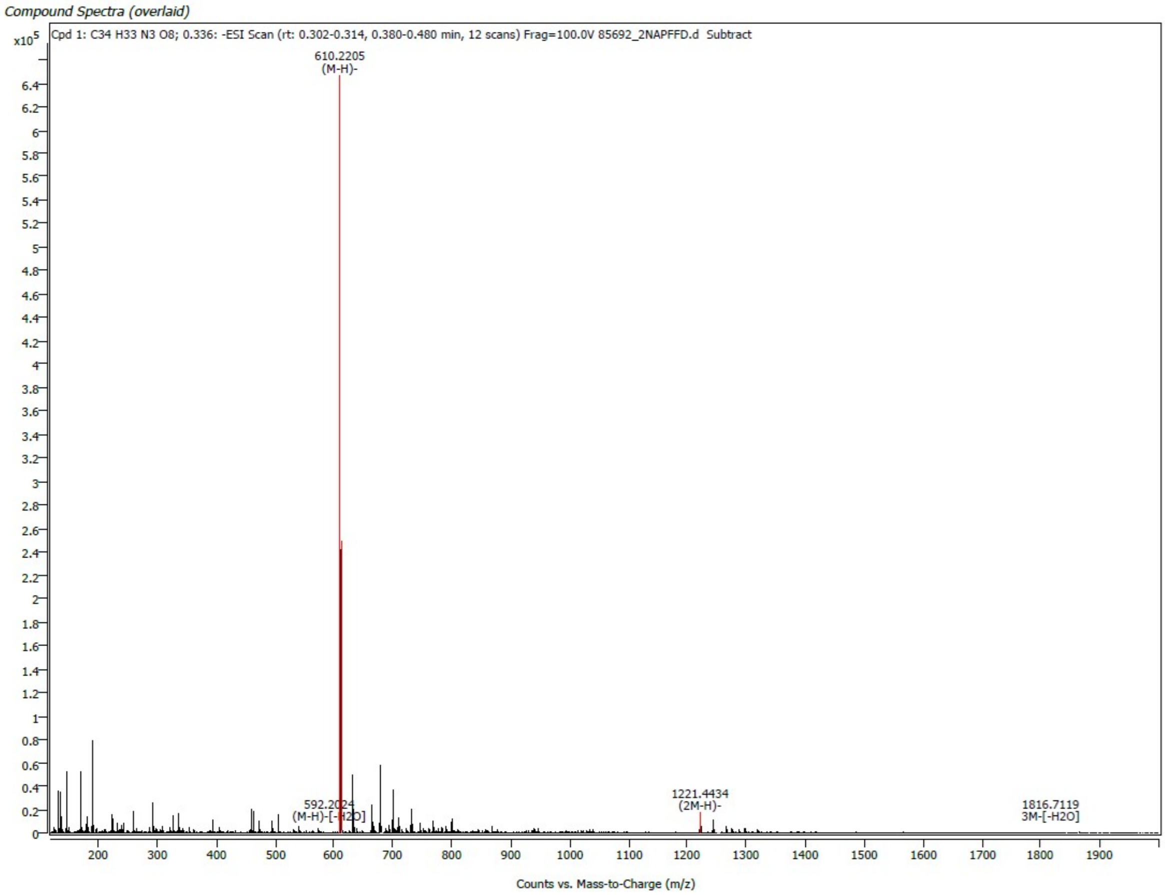


**Figure S3:** HRMS of *2NapFFD.*

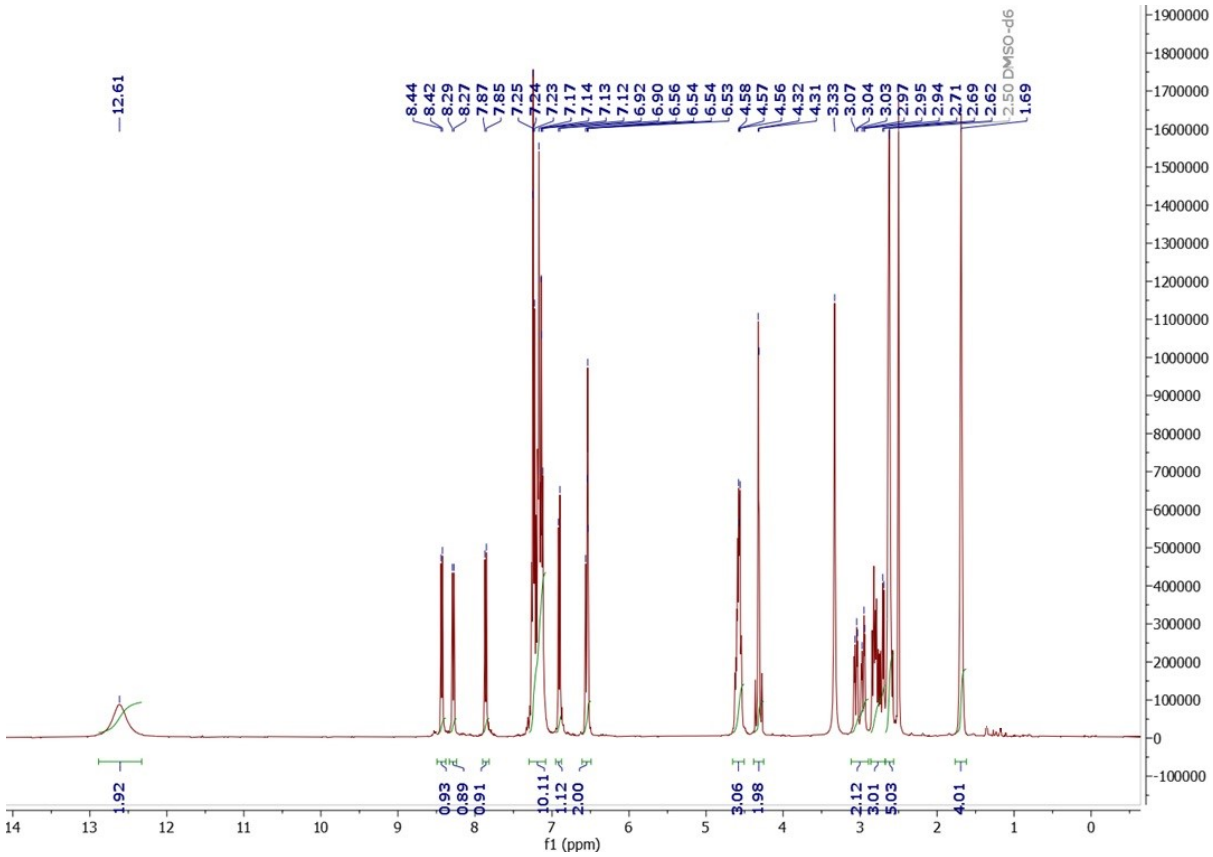

**Figure S4:** $^{1}$H-NMR of *2ThNapFFD*.

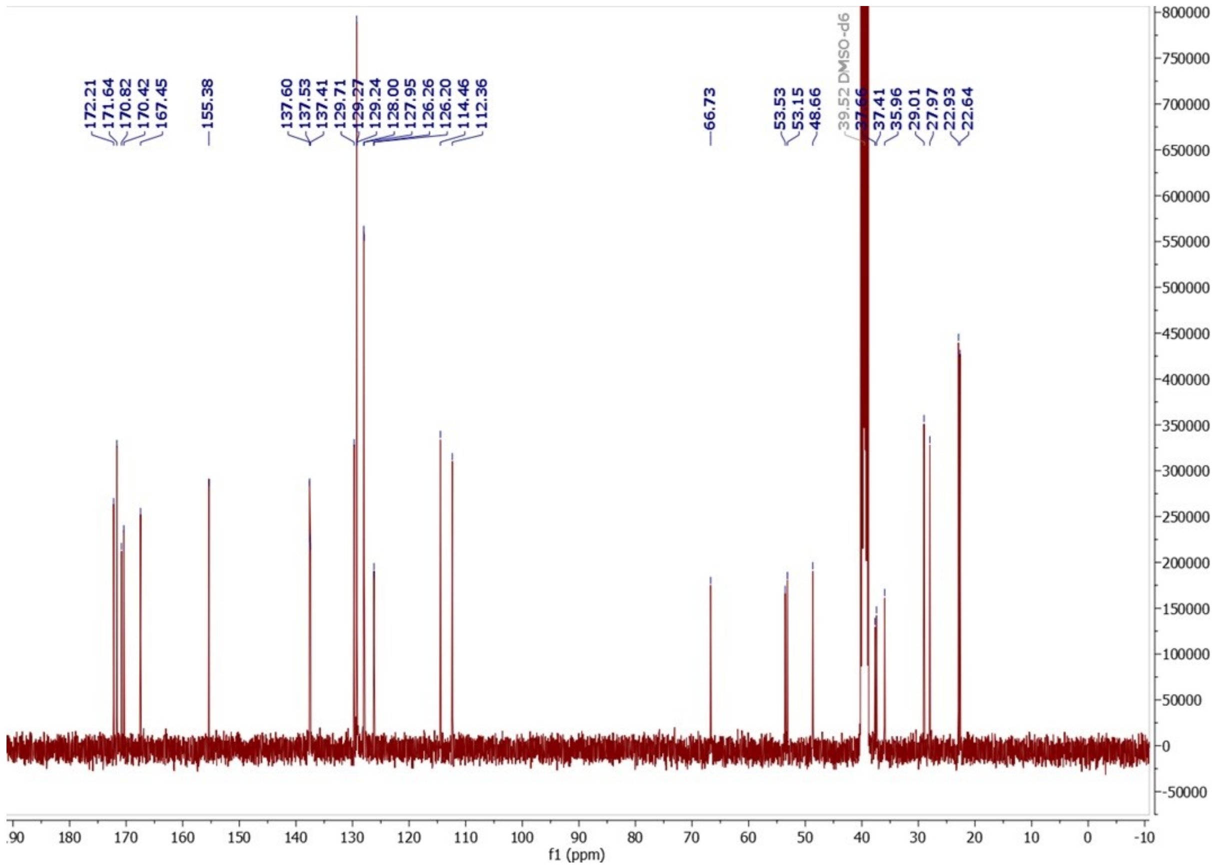

**Figure S5:** $^{13}$C-NMR of *2ThNapFFD*.

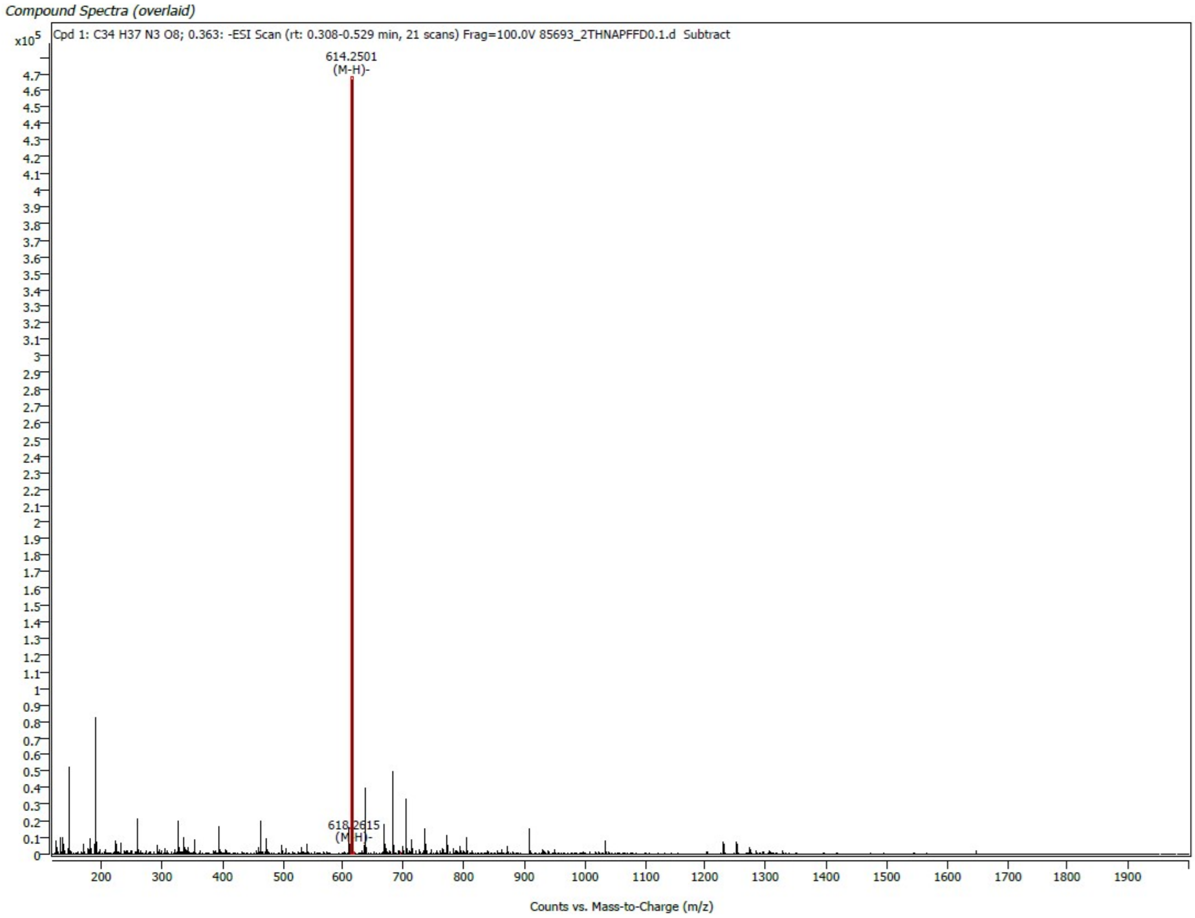


**Figure S6:** HRMS of *2ThNapFFD*.

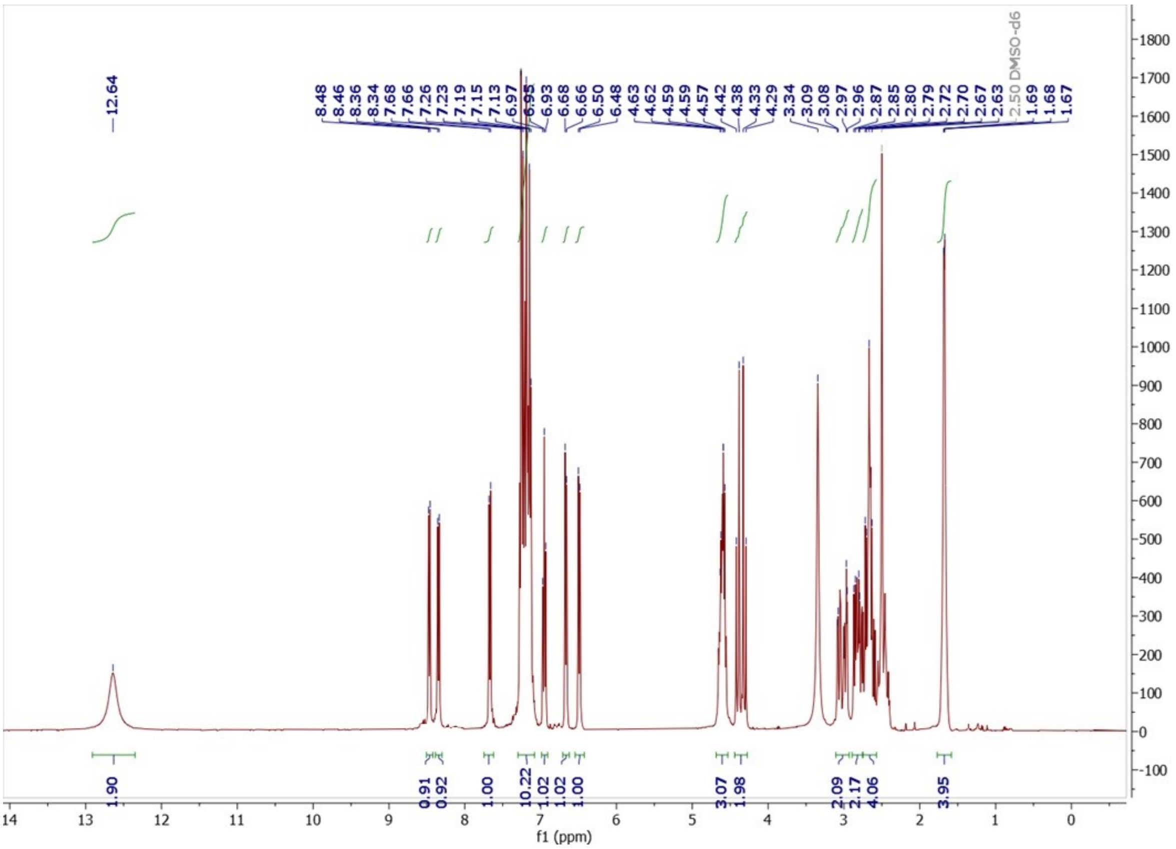

**Figure S7:** $^{1}$H-NMR of *1ThNapFFD*.

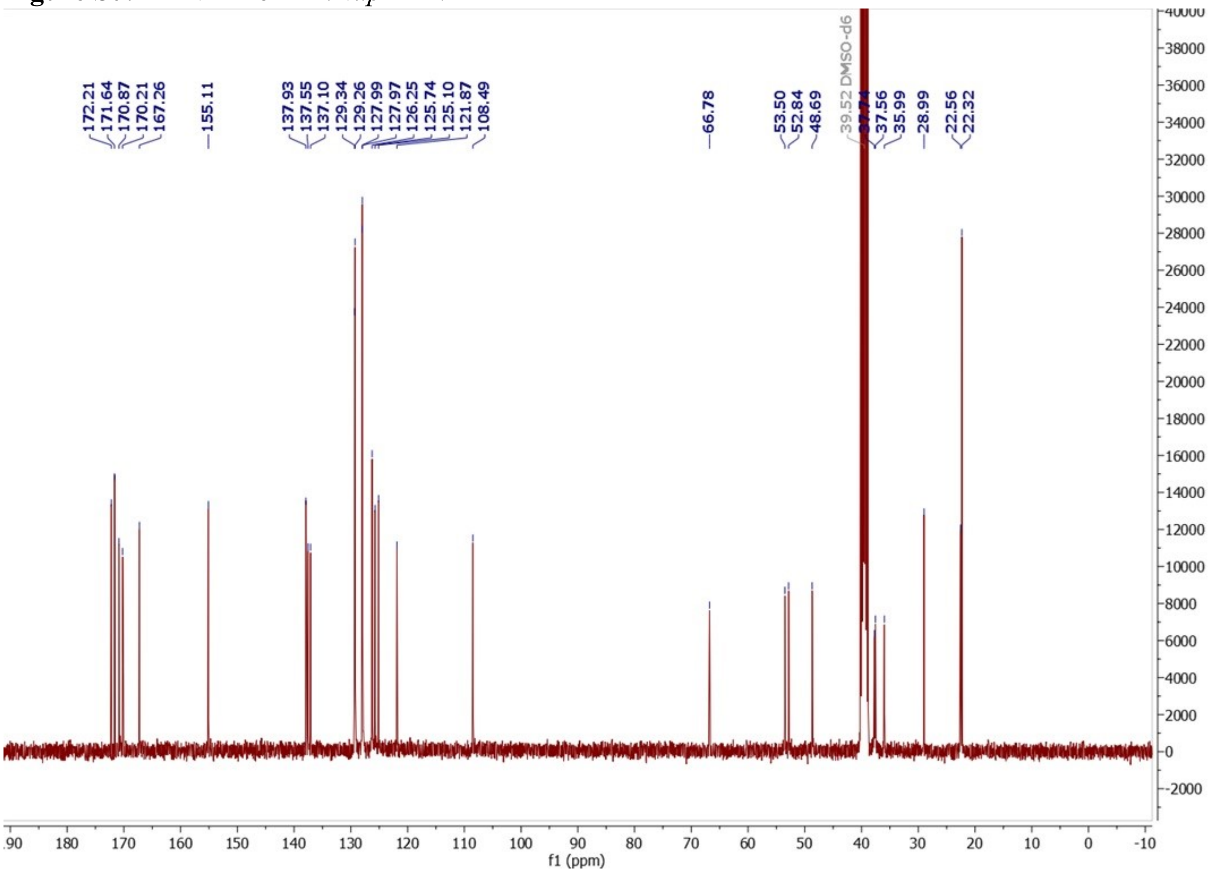


**Figure S8:** $^{13}$C-NMR of *1ThNapFFD*.

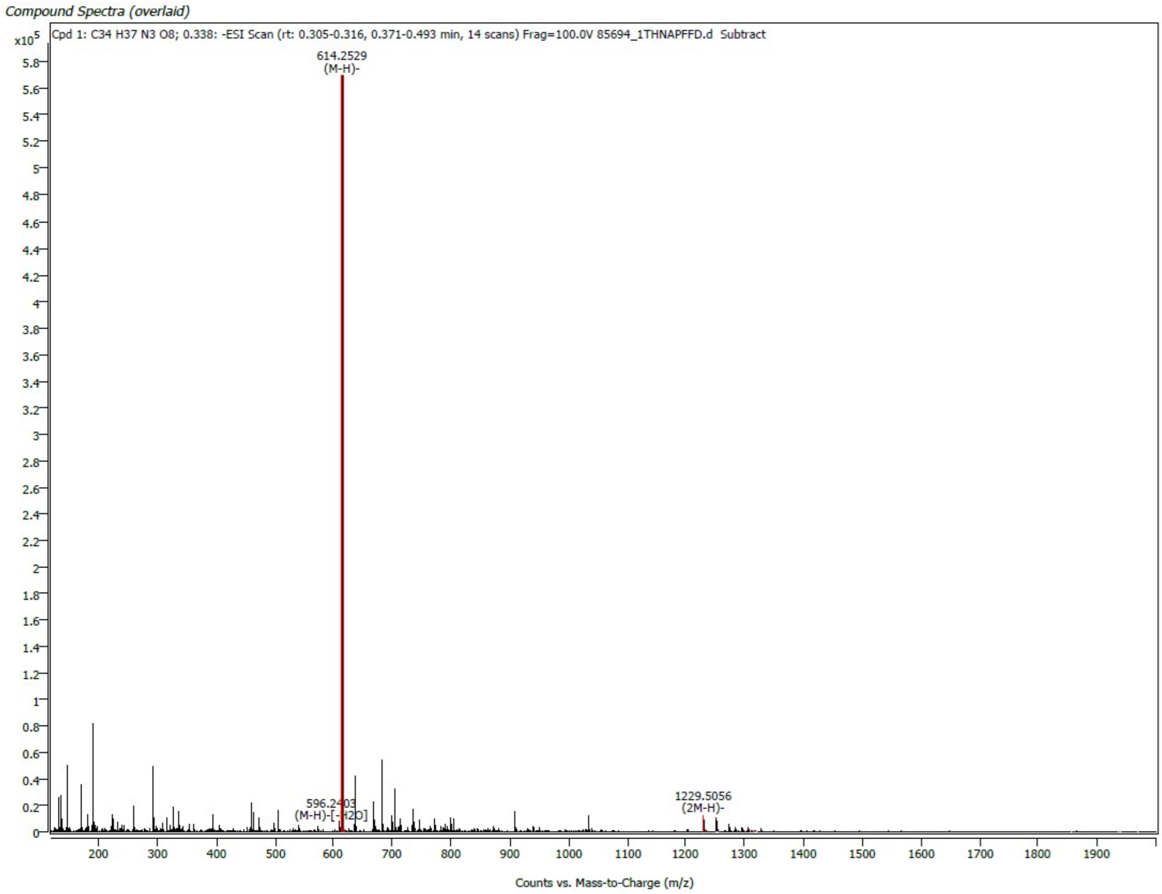

**Figure S9:** HRMS of *1ThNapFFD.*

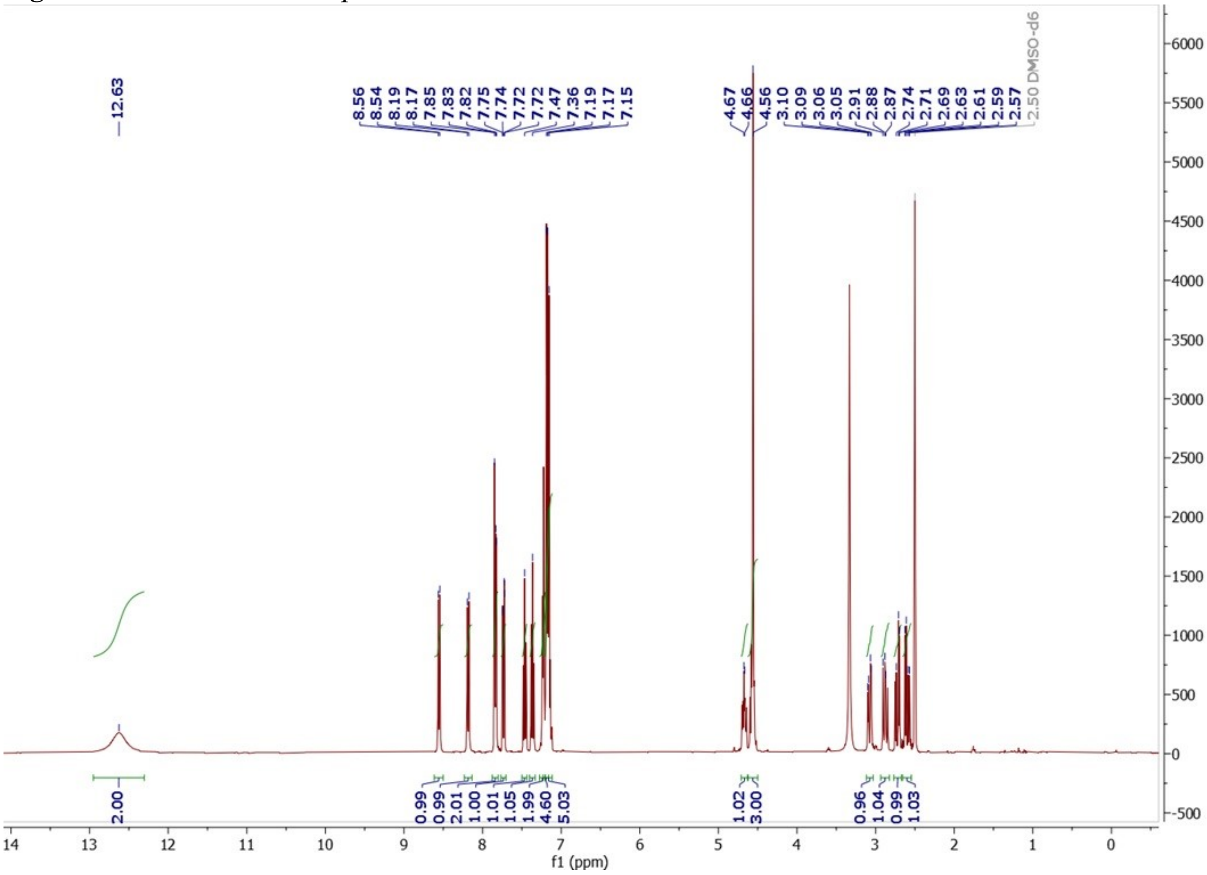


**Figure S10:** $^{1}$H-NMR of *2NapFD.*

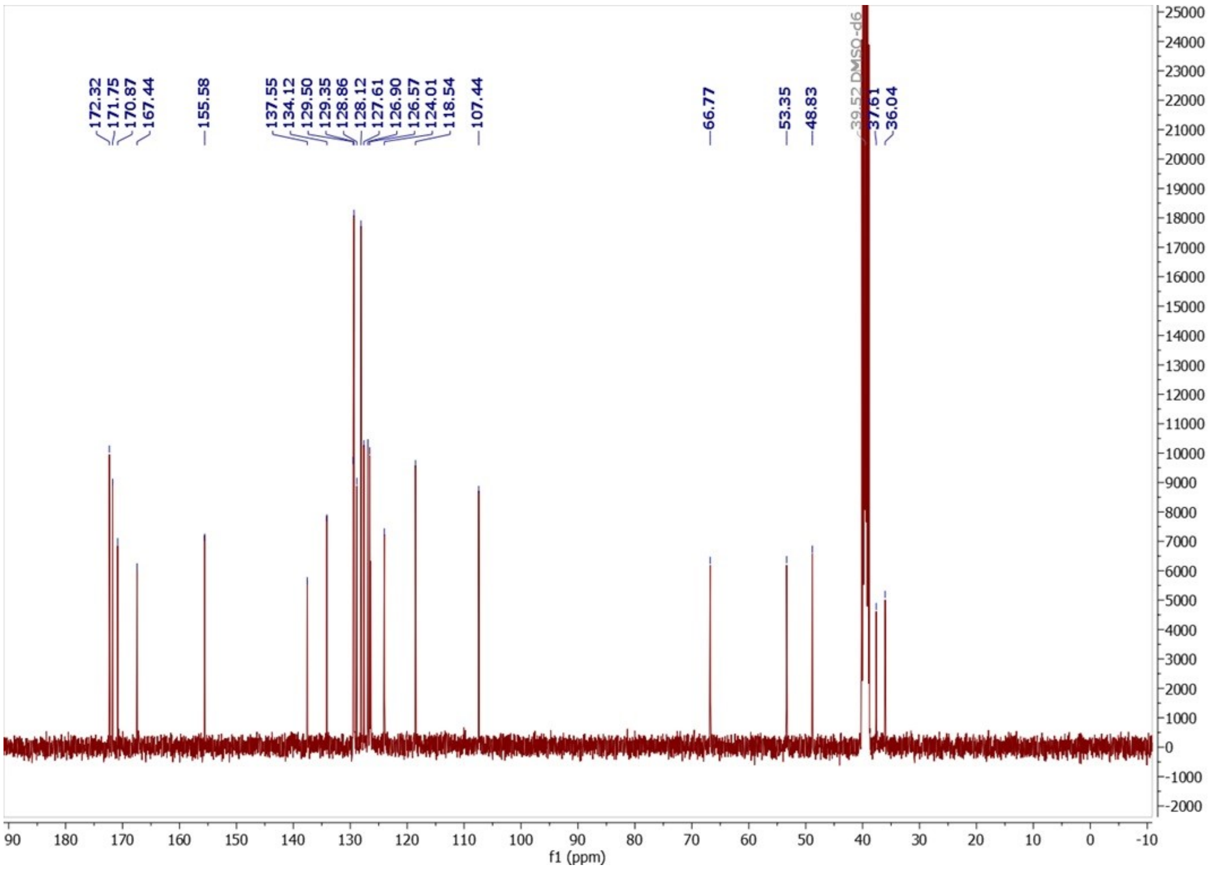

**Figure S11:** $^{13}$C-NMR of *2NapFD.*

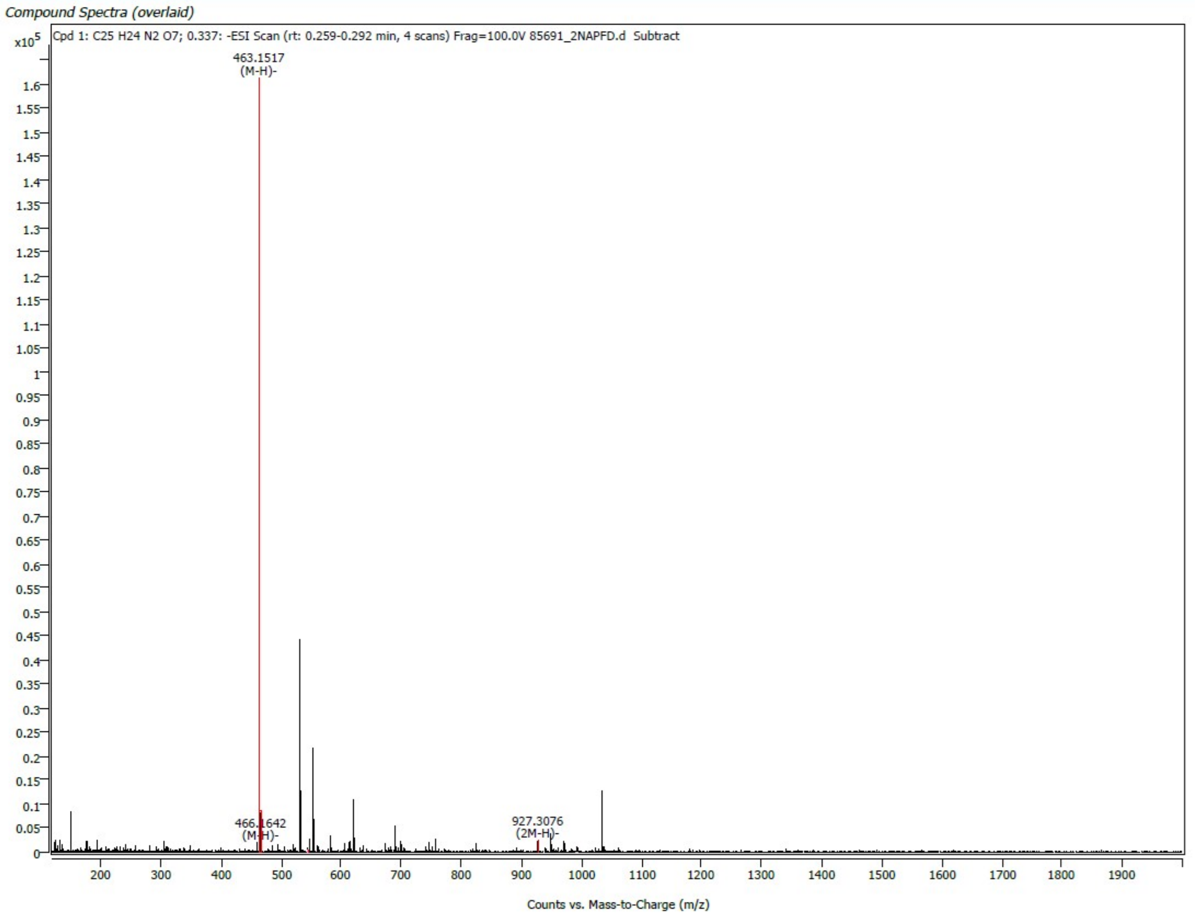


**Figure S12:** HRMS of *2NapFD.*

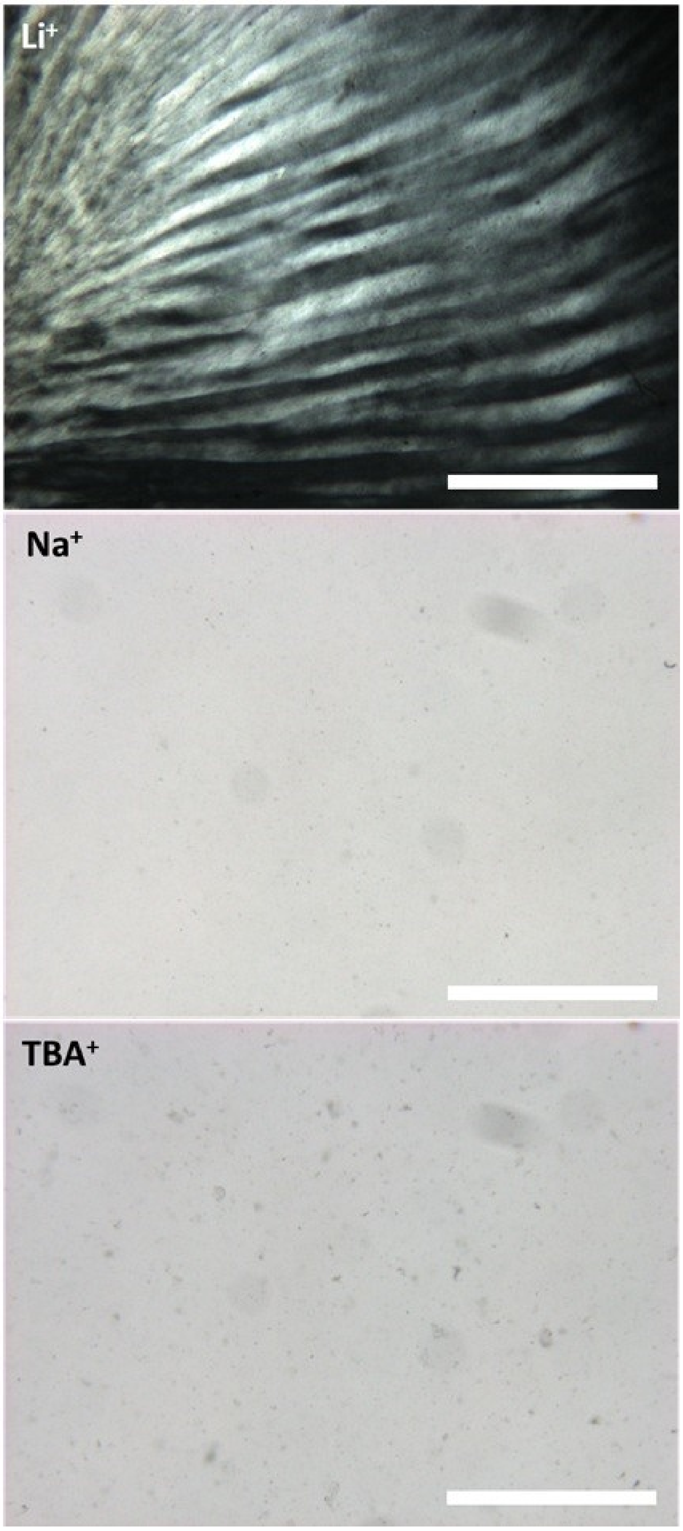


**Figure S13:** Microscopic images of 2NapFFD solutions prepared with $Li^+$, $Na^+$, and $TBA^+$ counterions (20 mg/mL, pH 10.5). The $Li^+$ sample is shown under polarised light (top), while the $Na^+$ and $TBA^+$ samples are brightfield images (middle and bottom). The polarised images of 2NapFFD·Na and 2NapFFD·TBA appeared completely black. Scale bars: 500 µm.

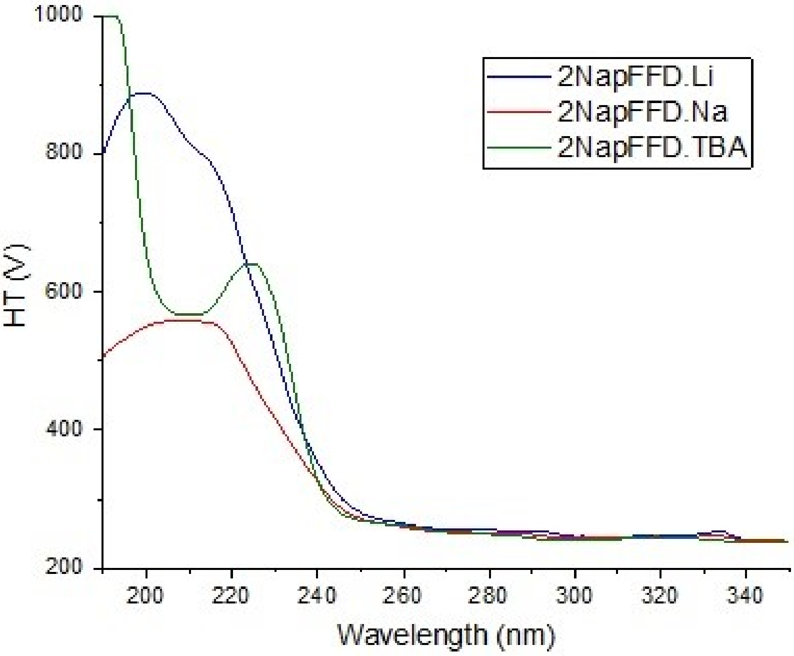


**Figure S14:** HT trace recorded during CD measurements of 2NapFFD solutions prepared with $Li^+$, $Na^+$, and $TBA^+$ counterions (20 mg/mL, pH 10.5).

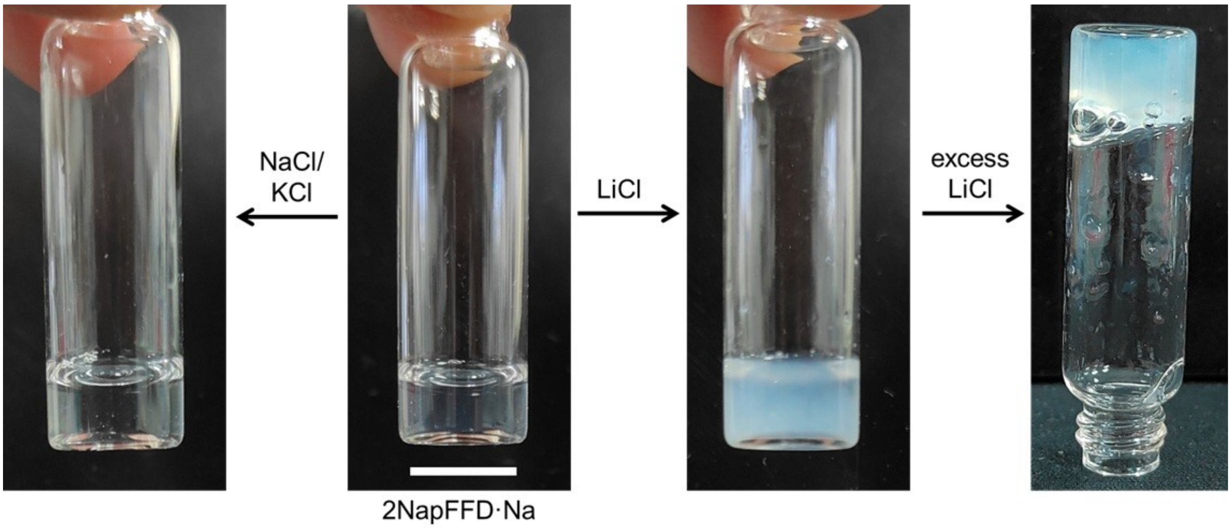


**Figure S15:** Photographs showing ion-selective response of 2NapFFD. Adding NaCl or KCl to 2NapFFD·Na solution (20 mg/mL, pH 10.5) produces no visible change, whereas adding LiCl induces significant thickening and further excess LiCl yields a self-supporting gel (tested by vial inversion). Scale bar: 1 cm.

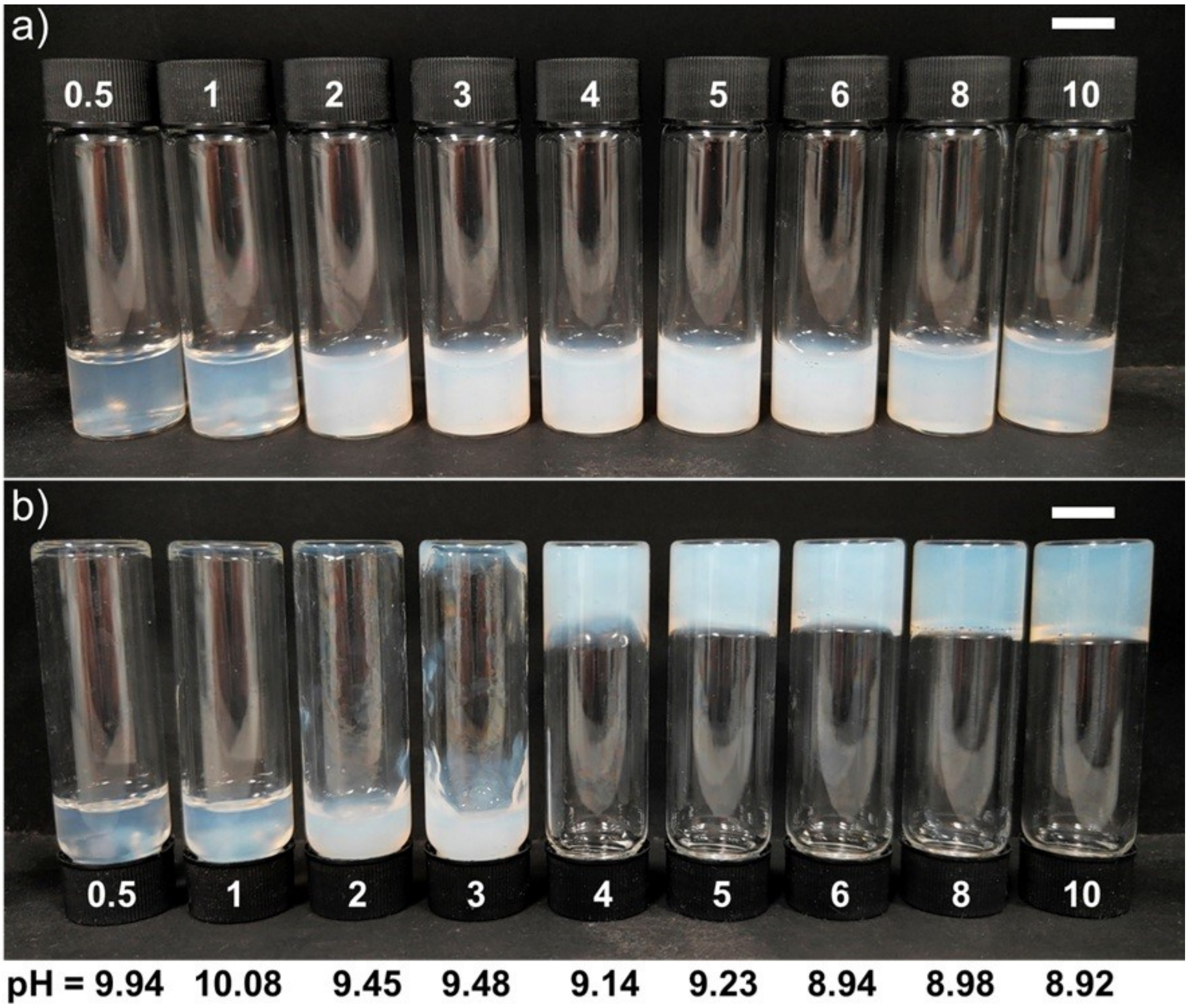


**Figure S16:** Photographs of 2NapFFD·Na solutions (20 mg/mL, pH 10.5) after addition of increasing equivalents of solid LiCl. The number of equivalents is shown on the vials. (a) Vials upright. (b) Vials after inversion, showing gel formation at higher LiCl equivalents. The final pH (after 24 h of mixing the LiCl) of the solutions are given below, showing basic pH for all the solutions. In the same time interval, the pH of the pure 2NapFFD·Na solution (without any added LiCl) drops to 10.28. Scale bars: 1 cm.

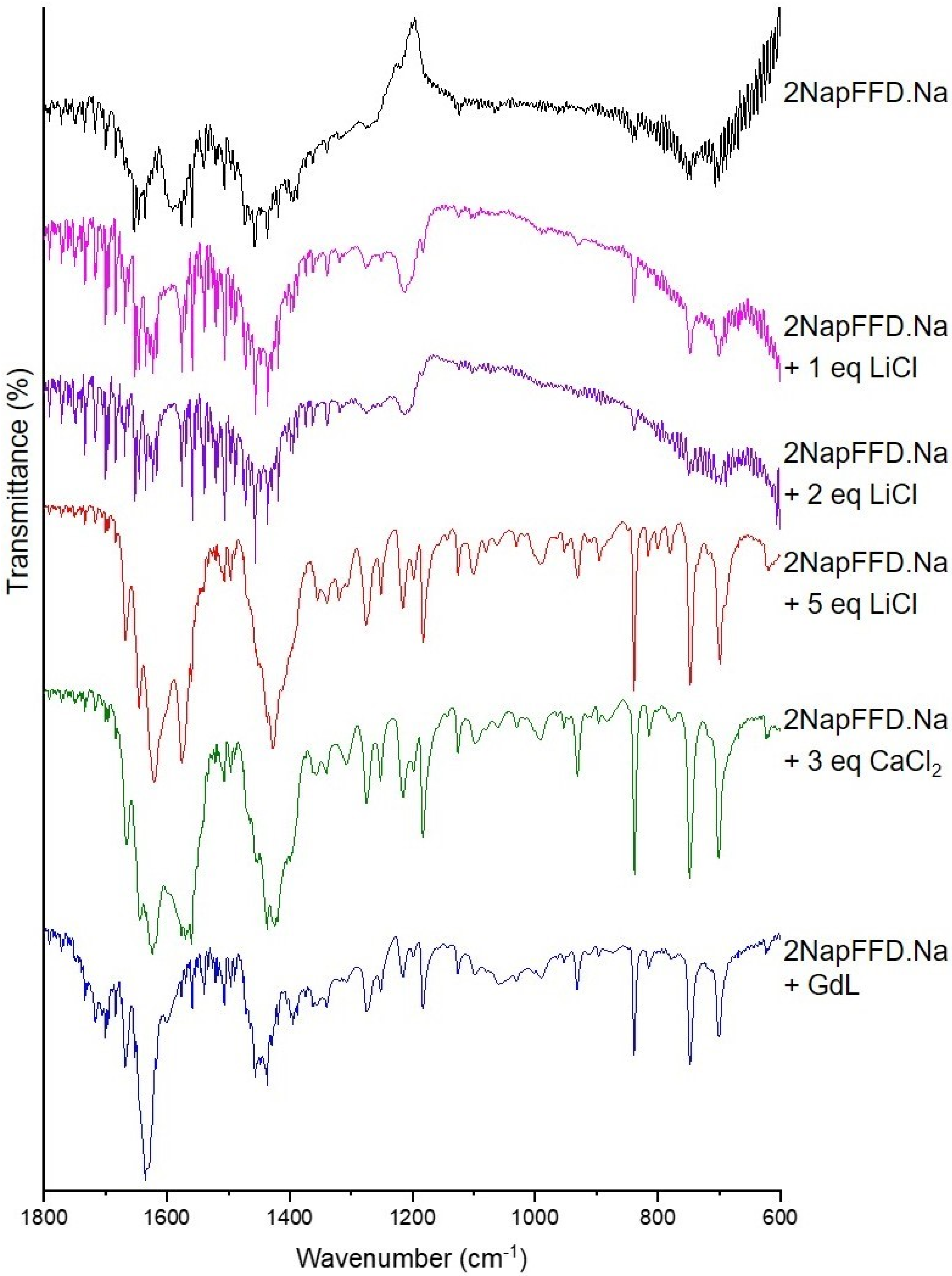


**Figure S17:** ATR-FTIR spectra recorded in $D_2O$ for 2NapFFD.Na salt solution, 2NapFFD.Na + LiCl (1 and 2 eq) solutions, and gels formed from 2NapFFD.Na by addition of 5 eq LiCl, 3 eq $CaCl_2$, or GdL. The LiCl- and $CaCl_2$-triggered gels show broadly similar spectral profiles, whereas the GdL-triggered gel shows a distinct profile, consistent with differences in protonation state and local hydrogen-bonding environment. The corresponding solution spectra were noisy, likely because liquid

samples in $D_2O$ give weaker ATR signals and less reproducible contact with the ATR crystal compared to the gel samples.

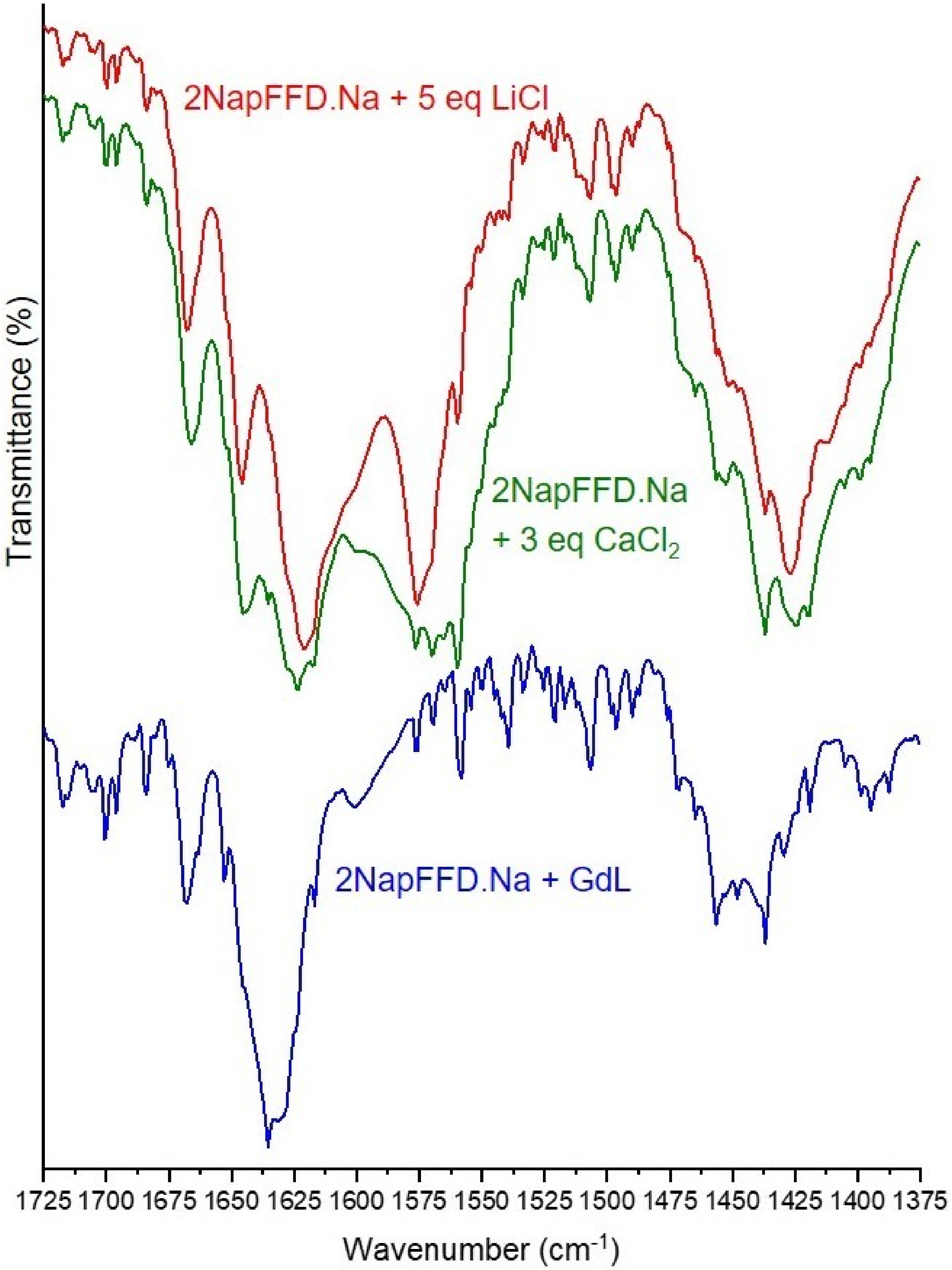


**Figure S18:** Expanded ATR-FTIR spectra of the amide/carboxylate region for gels formed from 2NapFFD.Na by addition of 5 eq LiCl, 3 eq $CaCl_2$, or GdL in $D_2O$.

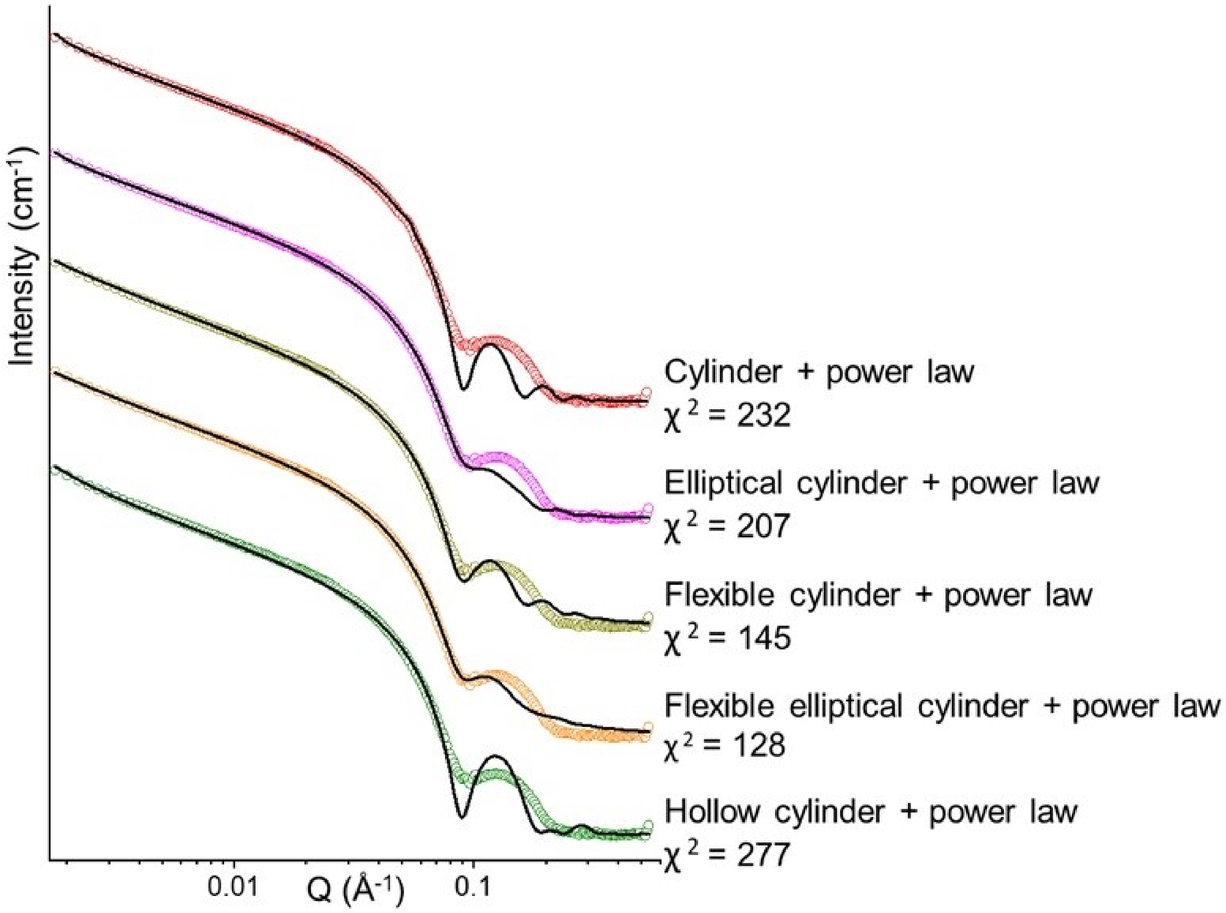


**Figure S19:** Fits obtained for 2NapFFD.Na (20 mg/mL, pD 10.9) + 2 eq LiCl with single cylindrical models and power law, corresponding $\chi^2$ values are provided adjacent to the fit. Similar fittings were obtained in all 2NapFFD/2ThNapFFD.Li samples.

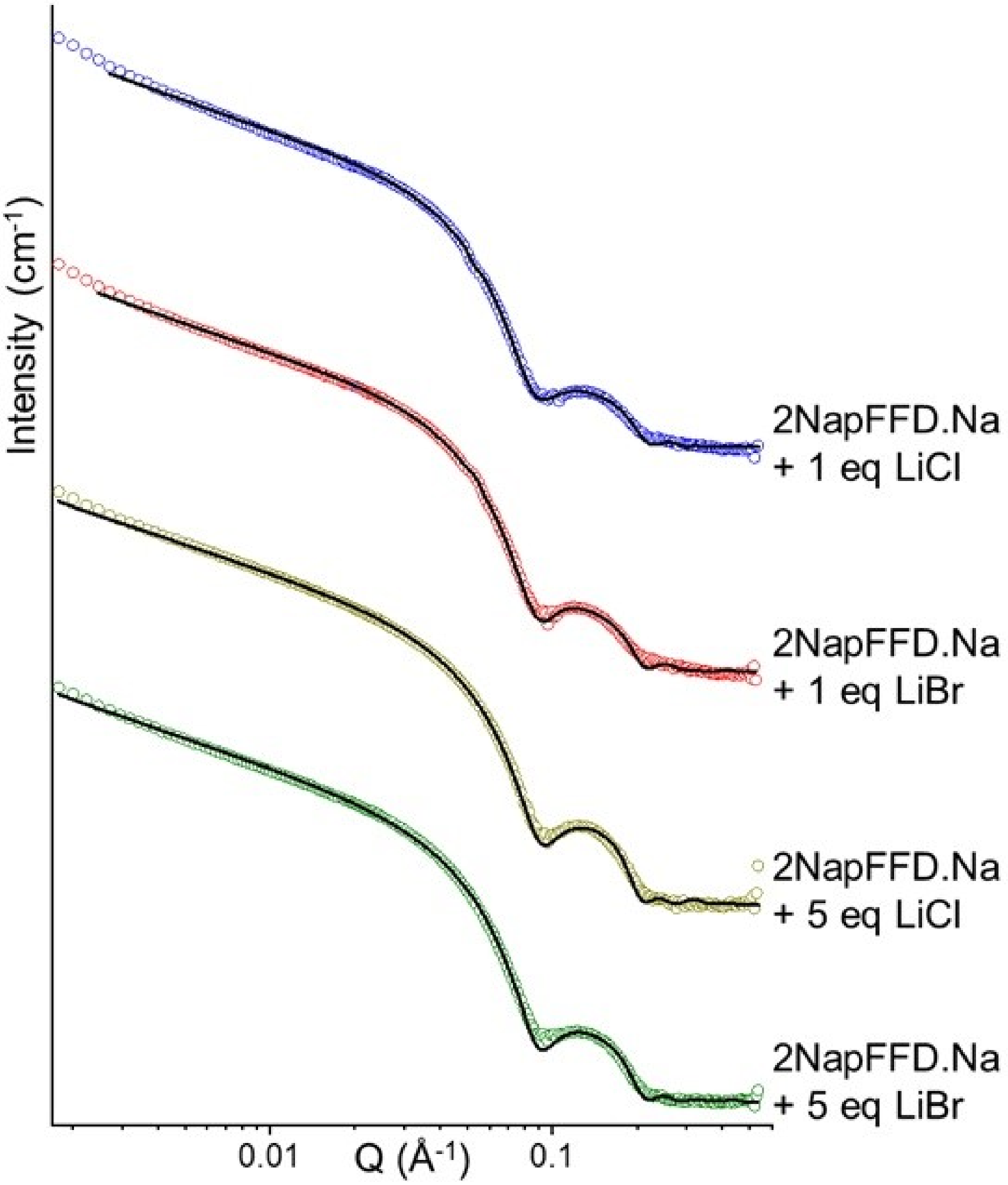


**Figure S20:** SANS profiles of 2NapFFD·Na (20 mg/mL, pD 10.9, $D_2O$) after adding 1 and 5 equivalents of LiCl and LiBr, showing similar scattering patterns.

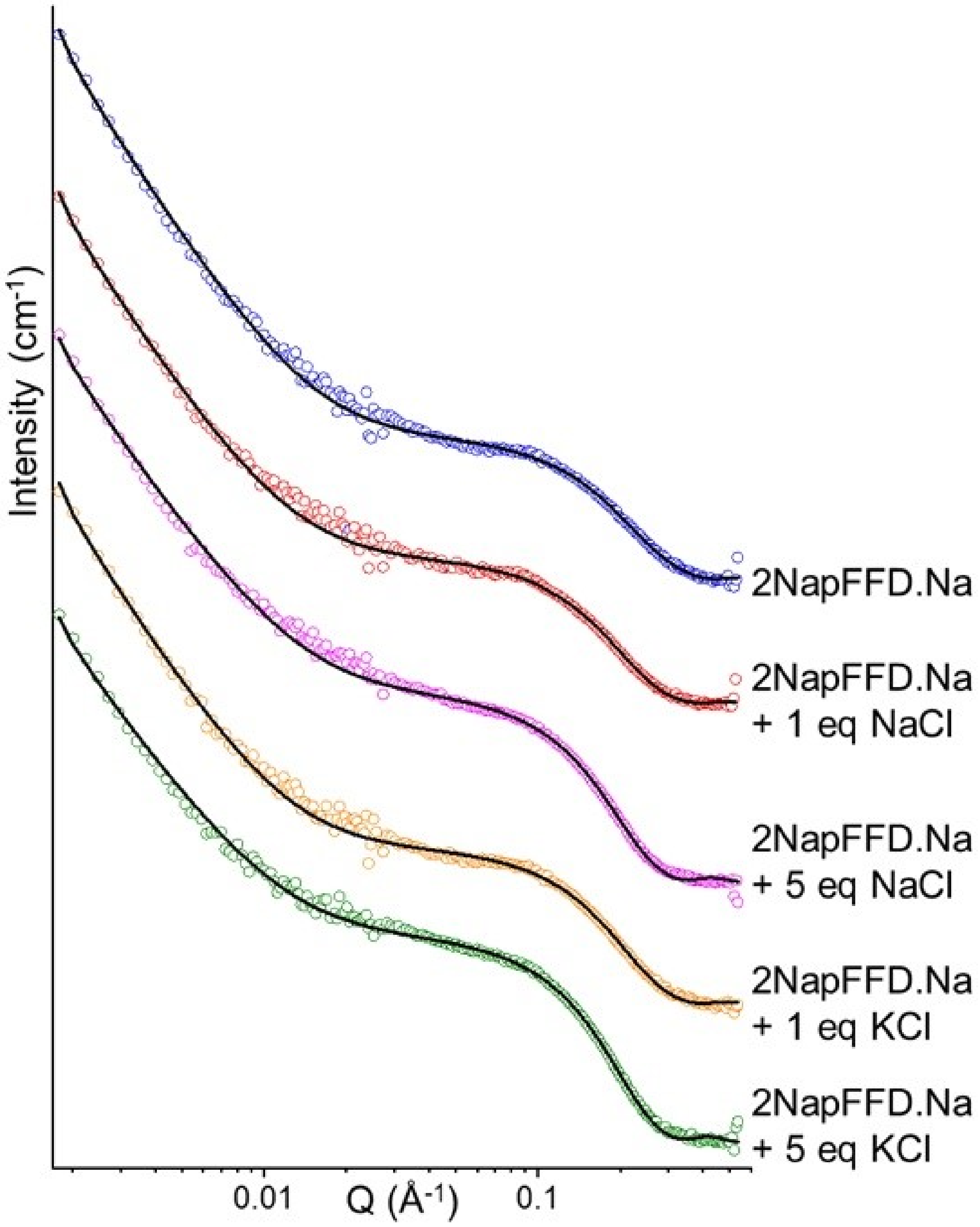


**Figure S21:** SANS profiles of 2NapFFD·Na (20 mg/mL, pD 10.9, $D_2O$) after adding 1 and 5 equivalents of NaCl and KCl, showing no change in scattering patterns.

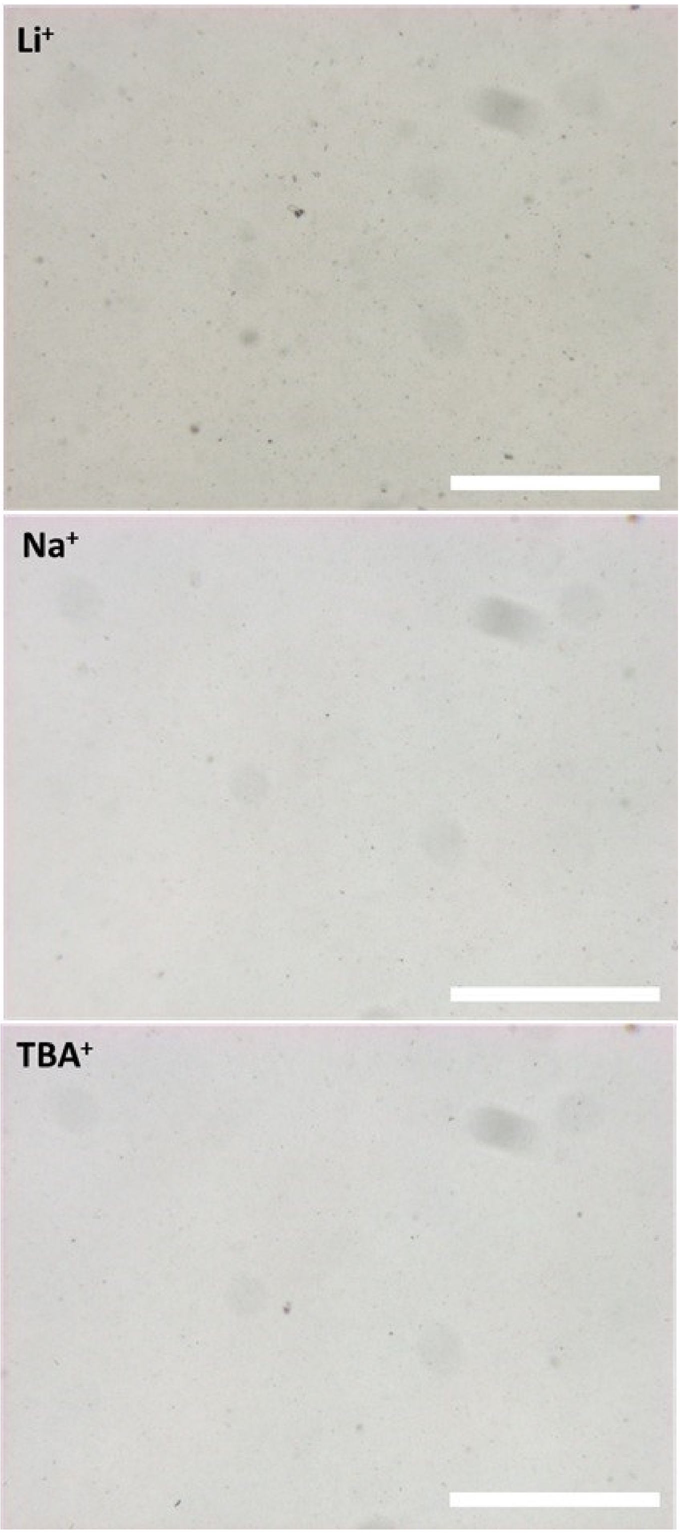


**Figure S22:** Brightfield optical micrographs of 2ThNapFFD solutions prepared with $Li^+$, $Na^+$, and $TBA^+$ counterions (20 mg/mL, pH 10.5). The polarised images of all the solutions appeared completely black. Scale bars: 500 μm.

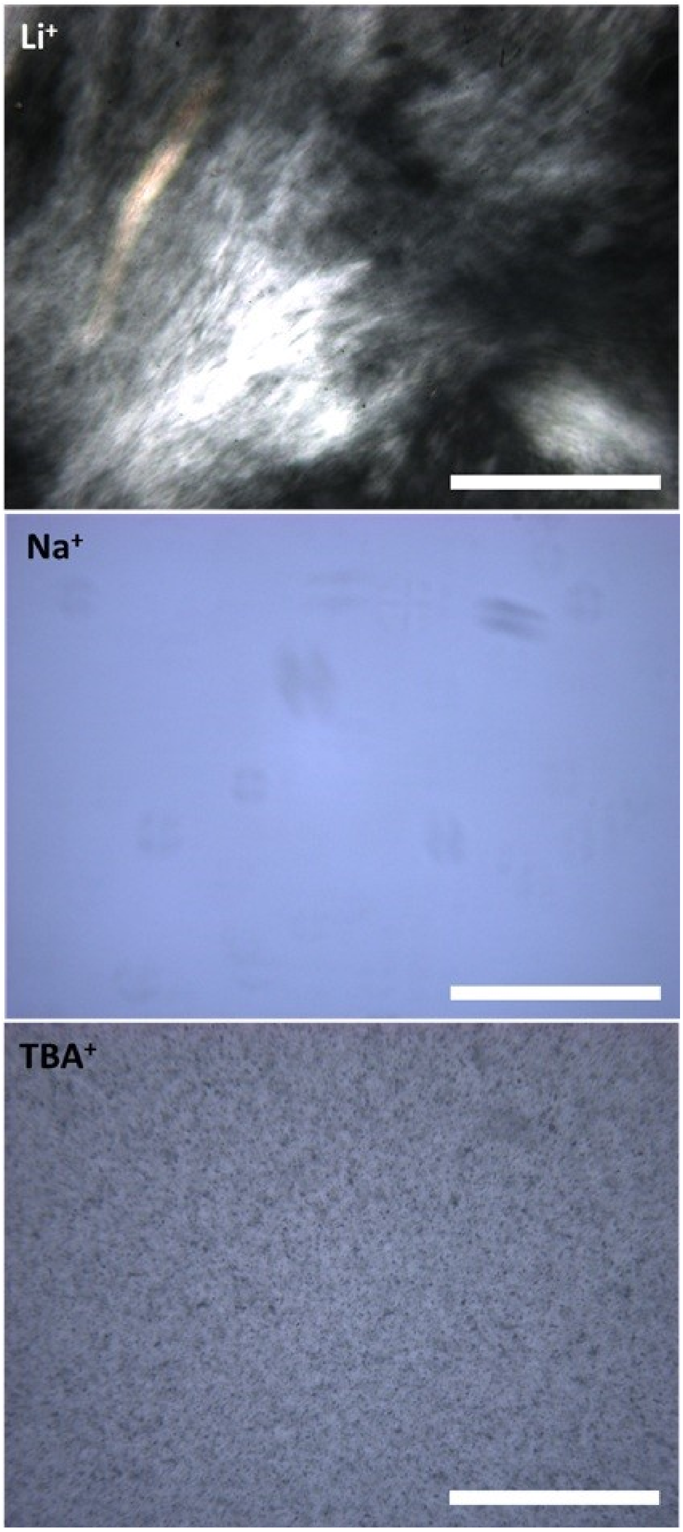


**Figure S23:** Microscopic images of 1ThNapFFD solutions prepared with $Li^+$, $Na^+$, and $TBA^+$ counterions (20 mg/mL, pH 10.5). The $Li^+$ sample is shown under polarised light (top), while the $Na^+$ and $TBA^+$ samples are brightfield images (middle and bottom). The polarised images of 1ThNapFFD·Na and 1ThNapFFD·TBA appeared completely black. Scale bars: 500 µm.

**Table S1.** Fitting parameters for SAXS data of 2NapFFD salts with counterions other than Li

| Counterions | Na | K | Rb | Cs | TBA | BTMA |
|---|---|---|---|---|---|---|
| Model | Cylinder + Power law | | | | | |
| Background ($cm^{-1}$) | 0.00043 ± 0.00021 | 0.0029 ± 0.0022 | 0.00625 ± 0.00086 | 0.01217 ± 0.00062 | 0.003 | 0.002 |
| A Scale | 0.00183 ± 0.00073 | 1.41 x$10^{-6}$ ± 4.07 x$10^{-7}$ | 0.00181 ± 0.00016 | 0.00226 ± 8.95 x$10^{-5}$ | 0.00127 ± 0.00010 | 0.00203 ± 9.12 x$10^{-5}$ |
| A Length (Å) | 46.4 ± 3.4 | 203 ± 22 | 50.9 ± 2.0 | 56.8 ± 1.3 | 26.1 ± 2.8 | 26.9 ± 1.8 |
| A Radius (Å) | 9.0 ± 1.7 | 7.2 ± 2.5 | 11.8 ± 0.6 | 13.3 ± 0.3 | 15.1 ± 3.3 | 15.6 ± 2.3 |
| B Scale | 2.82 x$10^{-6}$ ± 5.19 x$10^{-7}$ | 7.58 x$10^{-7}$ ± 3.44 x$10^{-7}$ | 4.89 x$10^{-7}$ ± 1.59 x$10^{-8}$ | 2.51 x$10^{-7}$ ± 2.36 x$10^{-9}$ | 2.82 x$10^{-8}$ ± 7.43 x$10^{-10}$ | 2.06 x$10^{-7}$ ± 2.30 x$10^{-9}$ |
| B Power | 2.55 ± 0.03 | 2.78 ± 0.08 | 3.28 ± 0.01 | 3.69 ± 0.00 | 3.89 ± 0.00 | 3.66 ± 0.00 |
| $\chi^2$ | 0.49 | 0.41 | 0.55 | 2.96 | 0.86 | 5.92 |

**Table S2.** Fitting parameters for SAXS data of 2NapFFD.Li solution

| | 2NapFFD.Li |
|---|---|
| Model | Cylinder + Hollow cylinder |
| Background ($cm^{-1}$) | 0.0052 ± 0.0003 |
| A Scale | 0.00242 ± 1.09 x$10^{-5}$ |
| A Length (Å) | >1000 |
| A Radius (Å) | 55.3 ± 0.1 |
| B Scale | 0.00183 ± 5.26 x$10^{-5}$ |
| B Length (Å) | 413 ± 9 |
| B Radius (Å) | 12.5 ± 0.0 |
| B Thickness (Å) | 20.2 ± 0.1 |
| $\chi^2$ | 4.34 |

**Table S3.** Fitting parameters for SAXS data of 2NapFFD solutions and GdL

| Counterions | Li | Na | TBA |
|---|---|---|---|
| Model | Elliptical cylinder + Power law | Elliptical cylinder | Cylinder + Power law |
| Background ($cm^{-1}$) | 0.005 | 0.0020 ± 0.0003 | 0.00778 ± 0.00048 |
| A Scale | 0.0070 ± 3.17 x$10^{-6}$ | 0.007 ± 1.06 x$10^{-5}$ | 0.00484 ± 1.6 x$10^{-5}$ |
| A Length (Å) | 766 ± 3 | >1000 | 144 ± 0.5 |
| A Radius (Å) | 45.1 ± 0.07 | 20.9 ± 0.03 | 32.0 ± 0.04 |
| A Axis ratio | 1.29 ± 0.004 | 5.52 ± 0.01 | - |
| B Scale | 2.48 x$10^{-11}$ ± 4.13 x$10^{-12}$ | - | 2.91 x$10^{-4}$ ± 1.69 x$10^{-5}$ |
| B Power | 4.95 ± 0.03 | - | 1.99 ± 0.01 |
| $\chi^2$ | 20.9 | 6.4 | 2.97 |

**Table S4.** Fitting parameters for SAXS data of 2NapFFD solutions with $CaCl_2$

| Counterions | Li | Na | TBA |
|---|---|---|---|
| Model | Flexible cylinder + Cylinder | Flexible elliptical cylinder | Flexible cylinder + Power law |
| Background ($cm^{-1}$) | 0.005 | 0.039 | 0.0052 |
| A Scale | 0.00324 ± 1.28 x$10^{-5}$ | 0.002298 ± 7.38 x$10^{-6}$ | 0.0055 ± 7.99 x$10^{-6}$ |
| A Length (Å) | >1000 | >1000 | >1000 |
| A Kuhn Length (Å) | 129 ± 0.3 | 134 ± 0.5 | 122 ± 0.4 |
| A Radius (Å) | 59.7 ± 0.1 | 29.1 ± 0.05 | 50.5 ± 0.03 |
| A Axis ratio | - | 2.33 ± 0.01 | - |
| B Scale | 0.00178 ± 1.39 x$10^{-5}$ | - | 3.51 x$10^{-5}$ ± 1.07 x$10^{-6}$ |
| B Length (Å) | >1000 | - | - |
| B Radius (Å) | 48.5 ± 0.1 | - | - |
| B Power | - | - | 2.75 ± 0.01 |
| $\chi^2$ | 2.7 | 5.8 | 30.0 |

**Table S5.** Fitting parameters for SANS data of 2NapFFD.Na-with added LiCl

| Eq of LiCl | 2NapFFD.Na | 2NapFFD.Na + 1 eq LiCl | 2NapFFD.Na + 2 eq LiCl | 2NapFFD.Na + 3 eq LiCl | 2NapFFD.Na + 4 eq LiCl | 2NapFFD.Na + 5 eq LiCl |
|---|---|---|---|---|---|---|
| Model | Cylinder + Power law | Cylinder + Hollow cylinder | | | | |
| Background ($cm^{-1}$) | 0.011 | 0.019 | 0.0197 ± 5.73 $x10^{-5}$ | 0.014 | 0.011 | 0.011 |
| A Scale | 0.017 ± 8.14 $x10^{-5}$ | 0.21 ± 0.001 | 0.0121 ± 7.68 $x10^{-5}$ | 0.12 ± 0.001 | 0.011 ± 0.0001 | 0.008 ± 9.2 $x10^{-5}$ |
| A Length (Å) | 30.3 ± 0.3 | >1000 | >1000 | >1000 | >1000 | >1000 |
| A Radius (Å) | 9.2 ± 0.04 | 46.6 ± 0.1 | 48.1 ± 0.1 | 48.4 ± 0.1 | 47.1 ± 0.07 | 46.6 ± 0.1 |
| B Scale | $3.0x10^{-6}$ ± 2.9 $x10^{-7}$ | 0.18 ± 0.001 | 0.0133 ± 7.67 $x10^{-5}$ | 0.013 ± 0.001 | 0.012 ± 0.0001 | 0.008 ± 9.2 $x10^{-5}$ |
| B Power | 2.59 ± 0.01 | - | - | - | - | - |
| B Length (Å) | - | 112 ± 1 | >1000 | >1000 | >1000 | >1000 |
| B Radius (Å) | - | 14.0 ± 0.07 | 11.2 ± 0.04 | 11.3 ± 0.05 | 11.3 ± 0.07 | 10.5 ± 0.05 |
| B Thick-ness (Å) | - | 20.1 ± 0.1 | 24.1 ± 0.1 | 23.9 ± 0.09 | 23.8 ± 0.1 | 25.2 ± 0.1 |
| $\chi^2$ | 2.27 | 30.28 | 13.7 | 12.6 | 12.1 | 15.9 |

**Table S6.** Fitting parameters for SANS data of 2NapFFD.Na-with added LiBr

| Sample | 2NapFFD.Na + 1 eq LiBr | 2NapFFD.Na + 5 eq LiBr |
|---|---|---|
| Model | Cylinder + Hollow cylinder | |
| Background ($cm^{-1}$) | 0.00845 ± 2.91 $x10^{-5}$ | 0.00862 ± 2.83 $x10^{-5}$ |
| A Scale | 0.00410 ± 1.89 $x10^{-5}$ | 0.00595 ± 4.01 $x10^{-5}$ |
| A Length (Å) | >1000 | >1000 |
| A Radius (Å) | 46.1 ± 0.1 | 47.2 ± 0.06 |
| B Scale | 0.00365 ± 2.05 $x10^{-5}$ | 0.00556 ± 4.12 $x10^{-5}$ |
| B Length (Å) | 117 ± 1 | >1000 |
| B Radius (Å) | 14.7 ± 0.07 | 11.9 ± 0.05 |
| B Thickness (Å) | 18.9 ± 0.1 | 22.5 ± 0.1 |
| $\chi^2$ | 11.3 | 17.5 |

**Table S7.** Fitting parameters for SANS data of 2NapFFD.Na-with added NaCl and KCl

| Sample | 2NapFFD.Na + 1 eq NaCl | 2NapFFD.Na + 5 eq NaCl | 2NapFFD.Na + 1 eq KCl | 2NapFFD.Na + 5 eq KCl |
|---|---|---|---|---|
| Model | Cylinder + Power law | | | |
| Background ($cm^{-1}$) | 0.0116 ± 3.52 $x10^{-5}$ | 0.00957 ± 3.23 $x10^{-5}$ | 0.00968 ± 3.45 $x10^{-5}$ | 0.00745 ± 3.18 $x10^{-5}$ |
| A Scale | 0.00700 ± 2.84 $x10^{-5}$ | 0.00789 ± 2.11 $x10^{-5}$ | 0.00680 ± 2.72 $x10^{-5}$ | 0.00747 ± 2.03 $x10^{-5}$ |
| A Length (Å) | 32.2 ± 0.2 | 36.7 ± 0.2 | 32.6 ± 0.2 | 37.6 ± 0.2 |
| A Radius (Å) | 10.4 ± 0.04 | 12.0 ± 0.03 | 10.5 ± 0.04 | 12.4 ± 0.03 |
| B Scale | 1.50 x $10^{-6}$ ± 1.73 $x10^{-7}$ | 5.48 x $10^{-6}$ ± 4.40 $x10^{-7}$ | 1.20 x $10^{-6}$ ± 1.44 $x10^{-7}$ | 5.66 x $10^{-6}$ ± 6.53 $x10^{-7}$ |
| B Power | 2.60 ± 0.02 | 2.45 ± 0.01 | 2.63 ± 0.02 | 2.36 ± 0.02 |
| $\chi^2$ | 2.04 | 3.06 | 2.47 | 2.52 |

**Table S8.** Fitting parameters for SAXS data of 2ThNapFFD.Li solution

| | 2ThNapFFD.Li |
|---|---|
| Model | Cylinder + Hollow cylinder |
| Background ($cm^{-1}$) | 0.00283 ± 1.02 x$10^{-4}$ |
| A Scale | 0.00292 ± 2.86 x$10^{-6}$ |
| A Length (Å) | >1000 |
| A Radius (Å) | 56.2 ± 0.1 |
| B Scale | 0.00199 ± 4.60 x$10^{-6}$ |
| B Length (Å) | >1000 |
| B Radius (Å) | 11.1 ± 0.0 |
| B Thickness (Å) | 20.6 ± 0.1 |
| $\chi^2$ | 29.8 |

**Table S9.** Fitting parameters for SAXS data of 2ThNapFFD salts with counterions other than Li

| Counterions | Na | K | Rb | Cs | TBA | BTMA |
|---|---|---|---|---|---|---|
| Model | Cylinder + Power law | | | | | |
| Background ($cm^{-1}$) | 0.004 | 0.005 | 0.005 | 0.01 | 0.003 | 0.0025 |
| A Scale | 0.000911 ± 1.84 x$10^{-5}$ | 0.000968 ± 1.99 x$10^{-5}$ | 0.0019 ± 2.87 x$10^{-5}$ | 0.0025 ± 3.17 x$10^{-5}$ | 0.001154 ± 7.43 x$10^{-5}$ | 0.001121 ± 7.42 x$10^{-5}$ |
| A Length (Å) | 725 ± 14 | 139 ± 4 | 777 ± 94 | 741 ± 72 | 29.4 ± 6.8 | 29.4 ± 14.8 |
| A Radius (Å) | 17.4 ± 0.2 | 17.5 ± 0.2 | 14.5 ± 0.1 | 13.9 ± 0.1 | 17.0 ± 0.8 | 17.0 ± 0.6 |
| B Scale | 1.38 x$10^{-8}$ ± 2.82 x$10^{-10}$ | 3.06 x$10^{-7}$ ± 6.75 x$10^{-9}$ | 3.89 x$10^{-7}$ ± 7.24 x$10^{-9}$ | 8.10 x$10^{-7}$ ± 9.80 x$10^{-9}$ | 8.82 x$10^{-8}$ ± 2.11 x$10^{-9}$ | 9.05 x$10^{-8}$ ± 2.25 x$10^{-9}$ |
| B Power | 3.65 ± 0.03 | 3.50 ± 0.00 | 3.47 ± 0.003 | 3.42 ± 0.002 | 3.67 ± 0.004 | 3.66 ± 0.004 |
| $\chi^2$ | 0.5 | 2.71 | 3.13 | 8.51 | 1.95 | 1.86 |

**Table S10.** Fitting parameters for SAXS data of 1ThNapFFD.Li solution

| | 1ThNapFFD.Li |
|---|---|
| Model | Elliptical Cylinder + Power law |
| Background ($cm^{-1}$) | 0.004 |
| A Scale | 0.00178 ± 2.20 x$10^{-6}$ |
| A Length (Å) | 322 ± 3 |
| A Radius (Å) | 45.2 ± 0.04 |
| A Axis ratio | 2.25 ± 0.003 |
| B Scale | 0.00118 ± 1.04 x$10^{-5}$ |
| B power | 1.9 ± 0.0017 |
| $\chi^2$ | 26.24 |

**Table S11.** Fitting parameters for SAXS data of 1ThNapFFD salts with counterions other than Li

| Counterions | Na | K | Rb | Cs | TBA | BTMA |
|---|---|---|---|---|---|---|
| Model | Cylinder + Power law | | | | | |
| Background ($cm^{-1}$) | 0.004 | 0.005 | 0.006 | 0.009 | 0.0025 | 0.003 |
| A Scale | 0.00134 ± 2.04 x$10^{-7}$ | 0.00101 ± 4.99 x$10^{-6}$ | 0.00245 ± 2.68 x$10^{-5}$ | 0.00262 ± 2.40 x$10^{-5}$ | 0.001324 ± 7.26 x$10^{-5}$ | 0.00128 ± 7.55 x$10^{-5}$ |
| A Length (Å) | 185 ± 5 | 277 ± 4 | 765 ± 67 | 771 ± 58 | 29.1 ± 2.7 | 29.2 ± 2.8 |
| A Radius (Å) | 17.1 ± 0.16 | 37.7 ± 0.10 | 14.9 ± 0.09 | 15.6 ± 0.1 | 17.0 ± 6.9 | 16.9 ± 8.6 |
| B Scale | 2.00 x$10^{-7}$ ± 1.54 x$10^{-8}$ | 1.88 x$10^{-5}$ ± 3.27 x$10^{-7}$ | 5.23 x$10^{-7}$ ± 9.0 x$10^{-9}$ | 7.55 x$10^{-7}$ ± 1.04 x$10^{-8}$ | 1.09 x$10^{-7}$ ± 8.44 x$10^{-9}$ | 8.64 x$10^{-8}$ ± 8.91 x$10^{-9}$ |
| B Power | 3.37 ± 0.01 | 2.83 ± 0.003 | 3.43 ± 0.003 | 3.40 ± 0.002 | 3.39 ± 0.014 | 3.38 ± 0.019 |
| $\chi^2$ | 0.67 | 8.05 | 3.93 | 7.11 | 0.63 | 0.59 |

**Table S12.** Fitting parameters for SANS data of 2ThNapFFD.Na-with added LiCl

| Eq of LiCl | 2ThNapFFD.Na | 2ThNapFFD.Na + 1 eq LiCl | 2ThNapFFD.Na + 2 eq LiCl | 2ThNapFFD.Na + 3 eq LiCl | 2ThNapFFD.Na + 4 eq LiCl | 2ThNapFFD.Na + 5 eq LiCl |
|---|---|---|---|---|---|---|
| Model | Cylinder + Power law | Cylinder + Hollow cylinder | | | | |
| Background ($cm^{-1}$) | 0.011 ± 3.3 $x10^{-5}$ | 0.00924 ± 2.87 $x10^{-5}$ | 0.00735 ± 2.84 $x10^{-5}$ | 0.0068 ± 2.86 $x10^{-5}$ | 0.0076 ± 2.87 $x10^{-5}$ | 0.0096 ± 2.9 $x10^{-5}$ |
| A Scale | 0.0074 ± 2.37 $x10^{-5}$ | 0.00369 ± 2.39 $x10^{-5}$ | 0.00521 ± 2.14 $x10^{-5}$ | 0.00611 ± 2.52 $x10^{-5}$ | 0.0057 ± 2.33 $x10^{-5}$ | 0.006 ± 2.4 $x10^{-5}$ |
| A Length (Å) | 69.2 ± 0.5 | >1000 | >1000 | >1000 | >1000 | >1000 |
| A Radius (Å) | 10.7 ± 0.02 | 47.4± 0.1 | 49.6 ± 0.1 | 48.1 ± 0.1 | 49.7 ± 0.1 | 49.1 ± 0.1 |
| B Scale | 4.7 $x10^{-6}$ ± 1.9 $x10^{-8}$ | 0.0068 ± 2.58 $x10^{-5}$ | 0.00695 ± 2.36 $x10^{-5}$ | 0.00675 ± 2.60 $x10^{-5}$ | 0.0073 ± 2.46 $x10^{-5}$ | 0.0073 ± 2.56 $x10^{-5}$ |
| B Power | 2.59 ± 0.01 | - | - | - | - | - |
| B Length (Å) | - | 121 ± 1 | 128 ± 1 | 130 ± 1 | 137 ± 1 | 129 ± 1 |
| B Radius (Å) | - | 13.7 ± 0.04 | 12.8 ± 0.04 | 12.9 ± 0.05 | 12.5 ± 0.03 | 12.7 ± 0.03 |
| B Thick-ness (Å) | - | 21.3 ± 0.1 | 22.1 ± 0.1 | 21.3 ± 0.1 | 22.1 ± 0.1 | 22.0 ± 0.1 |
| $\chi^2$ | 7.06 | 32.8 | 14.2 | 19.6 | 20.3 | 25.1 |

**Table S13.** Fitting parameters for SANS data of 1ThNapFFD.Na-with added LiCl

| Sample | 1ThNapFFD.Na | 1ThNapFFD.Na + 1 eq LiCl | 1ThNapFFD.Na + 2 eq LiCl | 1ThNapFFD.Na + 3 eq LiCl | 1ThNapFFD.Na + 4 eq LiCl | 1ThNapFFD.Na + 5 eq LiCl |
|---|---|---|---|---|---|---|
| Model | Cylinder + Power law | Elliptical cylinder + Power law | | | | |
| Background ($cm^{-1}$) | 0.0148 ± 3.44 $x10^{-5}$ | 0.0083 ± 3.12 $x10^{-5}$ | 0.00872 ± 2.94 $x10^{-5}$ | 0.0065 ± 3.32 $x10^{-5}$ | 0.0173 ± 6.54 $x10^{-5}$ | 0.0119 ± 6.54 $x10^{-5}$ |
| A Scale | 0.0146 ± 3.77 $x10^{-5}$ | 0.00995 ± 1.97 $x10^{-5}$ | 0.0102 ± 1.40 $x10^{-5}$ | 0.0101 ± 1.97 $x10^{-5}$ | 0.0194 ± 3.63 $x10^{-5}$ | 0.0187 ± 3.73 $x10^{-5}$ |
| A Length (Å) | 38.2 ± 0.2 | >1000 | >1000 | 133 ± 1 | 117 ± 1 | 128 ± 1 |
| A Radius (Å) | 10.9 ± 0.02 | 10.6 ± 0.03 | 13.8 ± 0.02 | 17.2 ± 0.03 | 18.4 ± 0.03 | 17.1 ± 0.03 |
| A Axis ratio | - | 3.07 ± 0.01 | 2.34 ± 0.01 | 2.02 ± 0.01 | 1.96 ± 0.01 | 2.02 ± 0.01 |
| B Scale | 6.19$x10^{-6}$ ± 8.17 $x10^{-8}$ | 5.42 $x10^{-6}$ ± 1.26 $x10^{-7}$ | 3.52 $x10^{-6}$ ± 9.12 $x10^{-8}$ | 7.60 $x10^{-5}$ ± 1.39 $x10^{-6}$ | 1.53 $x10^{-4}$ ± 2.48 $x10^{-6}$ | 0.000145 ± 2.65 $x10^{-6}$ |
| B Power | 2.81 ± 0.00 | 2.83 ± 0.04 | 2.89 ± 0.04 | 2.40 ± 0.03 | 2.41 ± 0.02 | 2.40 ± 0.03 |
| $\chi^2$ | 28.0 | 30.28 | 29.5 | 9.83 | 12.1 | 8.59 |

**Table S14.** Fitting parameters for SAXS data of 2NapFD solutions

| Counterions | Li | Na | TBA |
|---|---|---|---|
| Model | Cylinder + Power law | | |
| Background ($cm^{-1}$) | 0.004 | 0.009 | 0.01 |
| A Scale | 0.00464 ± 3.45 x$10^{-5}$ | 0.00234 ± 2.90 x$10^{-5}$ | 0.00051 ± 0.00018 |
| A Length (Å) | 62.2 ± 0.2 | 36.7 ± 1.0 | 32.9 ± 0.1 |
| A Radius (Å) | 42.7 ± 0.1 | 9.6 ± 0.9 | 9.9 ± 0.0 |
| B Scale | 3.26 x$10^{-6}$ ± 4.27 x$10^{-8}$ | 1.33 x$10^{-6}$ ± 9.65 x$10^{-8}$ | 1.37 x$10^{-8}$ ± 1.54 x$10^{-9}$ |
| B Power | 3.11 ± 0.00 | 2.86 ± 0.01 | 3.72 ± 0.20 |
| $\chi^2$ | 4.45 | 0.40 | 0.38 |

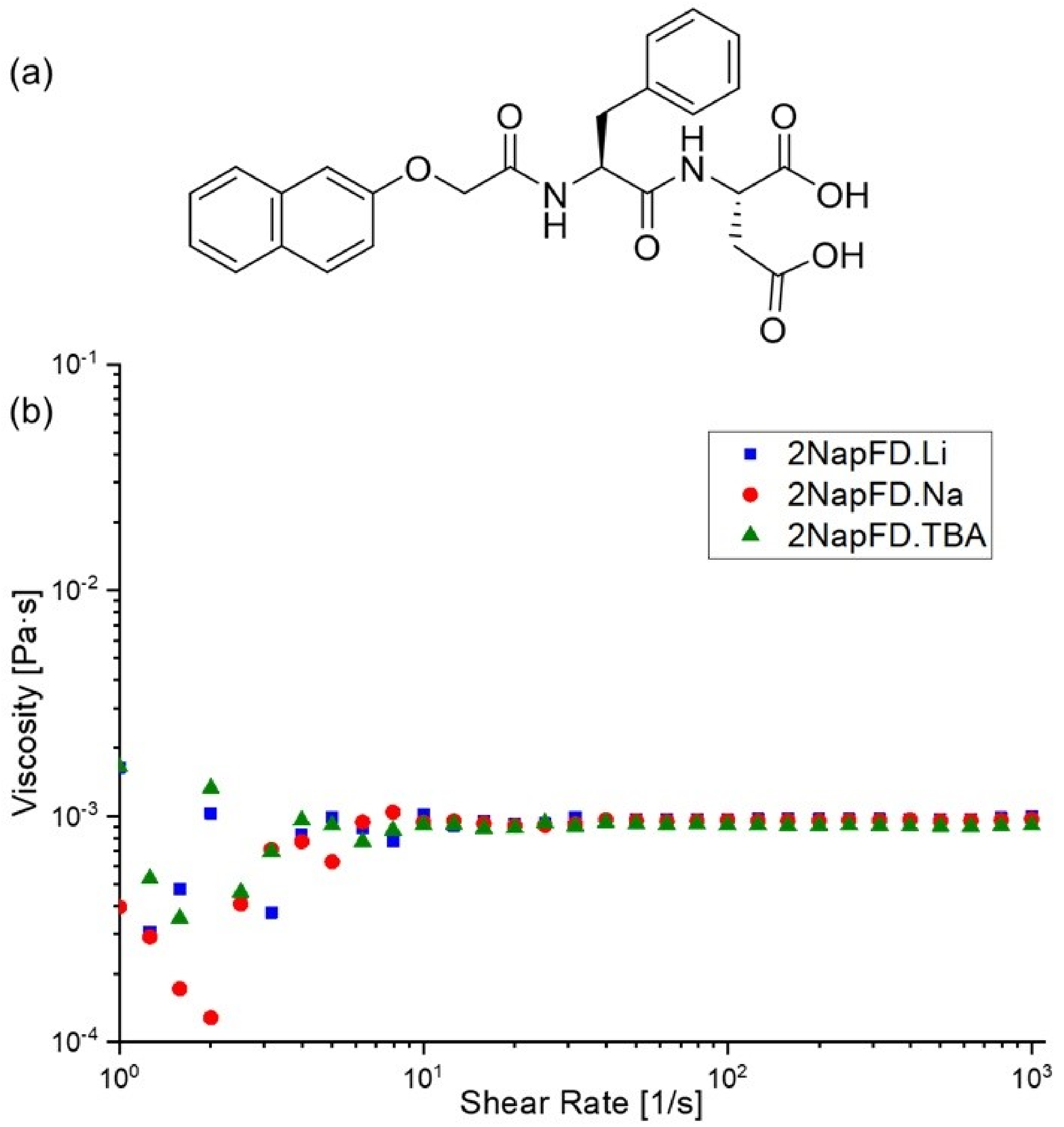


**Figure S24:** (a) Chemical structure of 2NapFD. (b) Viscosity profiles of its $Li^+$, $Na^+$, and $TBA^+$ salts (20 mg/mL, pH 10.5) measured as a function of shear rate (bottom). All samples exhibit low viscosities comparable to water, indicating the absence of significant self-assembly.

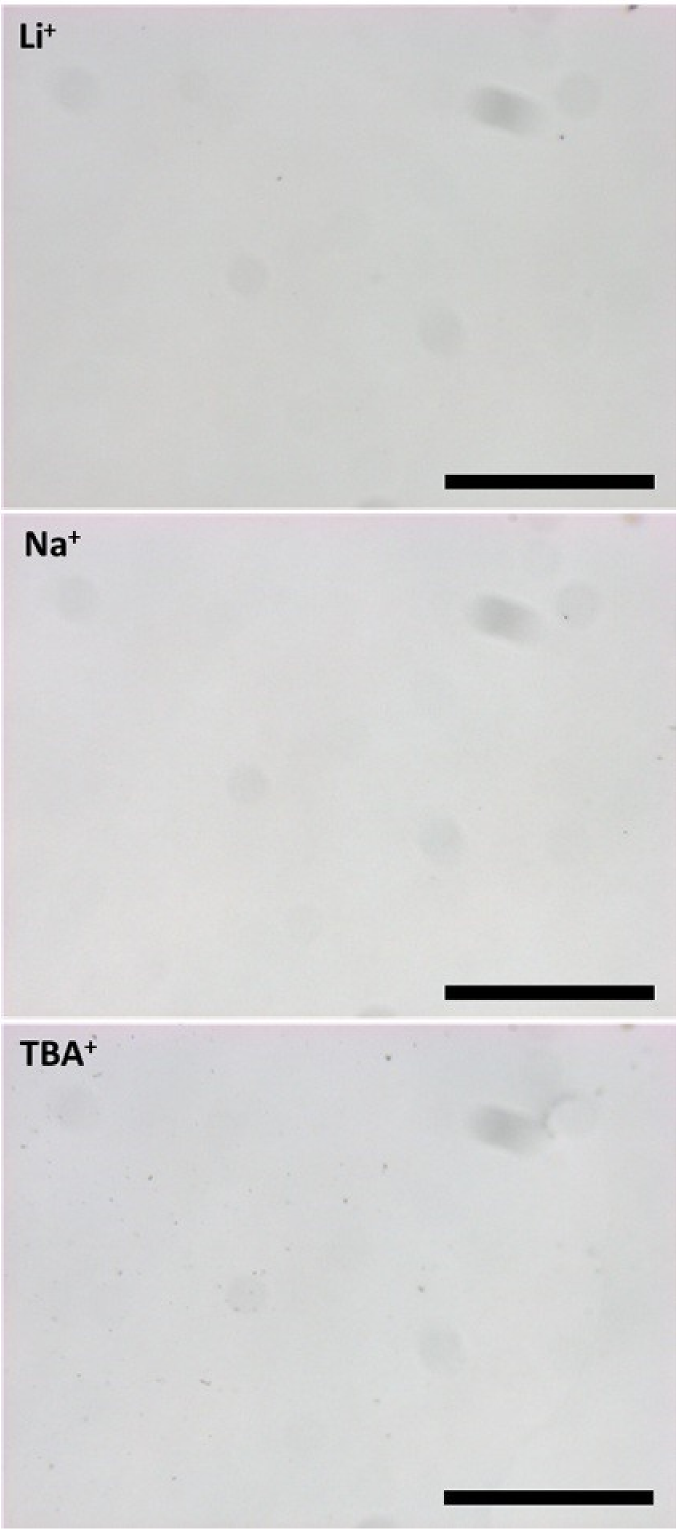


**Figure S25:** Brightfield optical micrographs of 2NapFD solutions prepared with $Li^+$, $Na^+$, and $TBA^+$ counterions (20 mg/mL, pH 10.5). The polarised images of all the solutions appeared completely black. Scale bars: 500 µm.

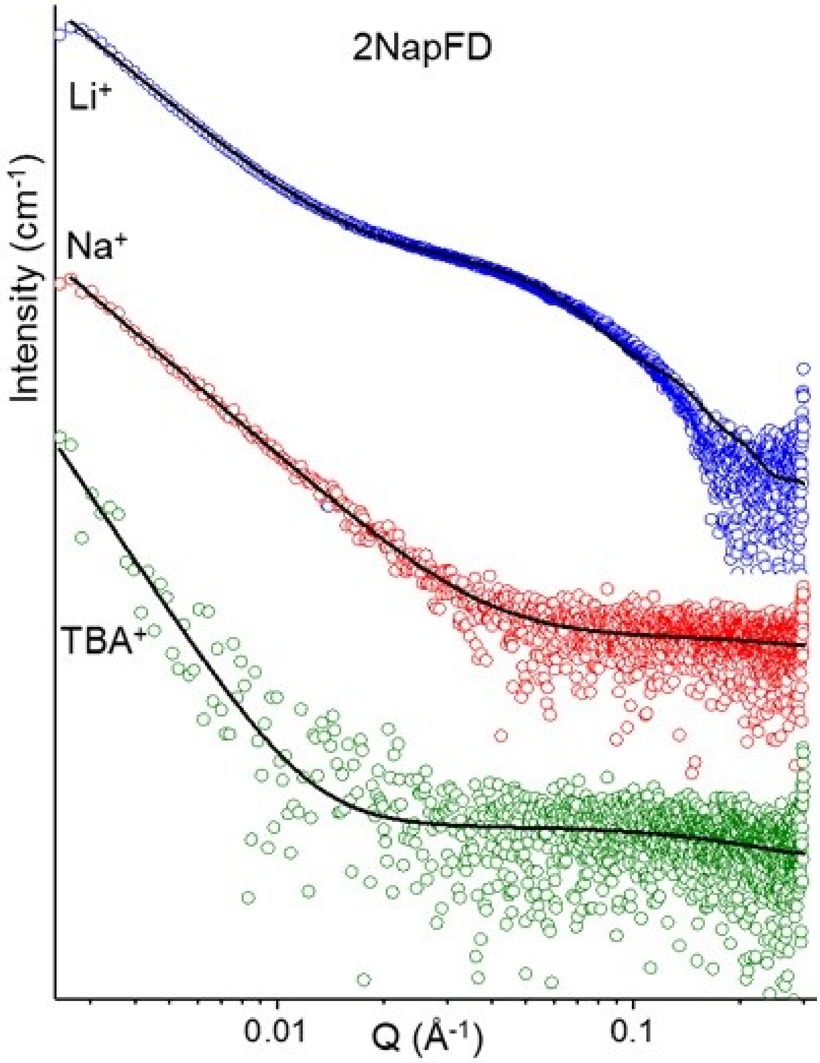


**Figure S26:** SAXS profiles of 2NapFD prepared with different counterions ($Li^+$, $Na^+$, and $TBA^+$) at pH 10.5 and 20 mg/mL. Symbols show the experimental data and solid lines show the best fits (power law + cylinder model), indicating only short, weakly aggregated structures across all three salts.

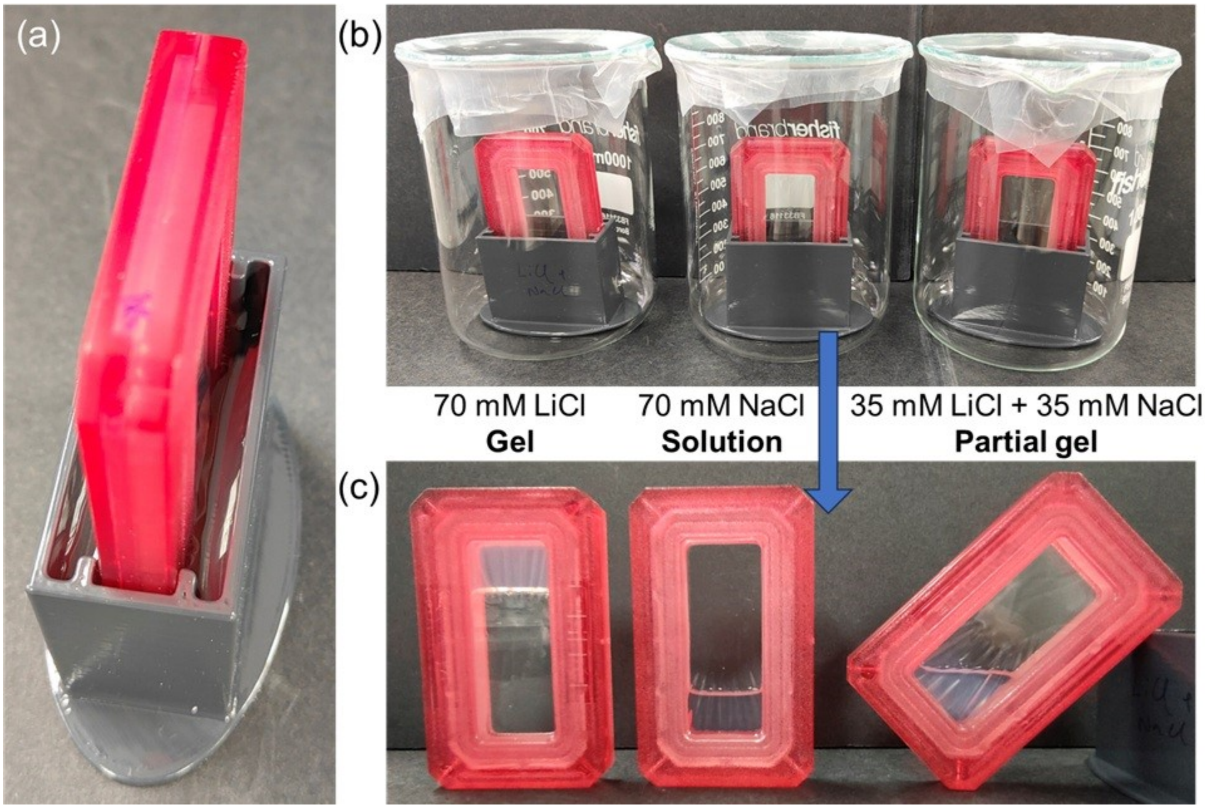


**Figure S27:** Dialysis setup used for ICP-OES quantification of ion uptake. (a) Custom 3D-printed cassette holder to minimize NaCl/LiCl dialysate volume. (b) Dialysis set up using 70 mM LiCl, 70 mM NaCl, or 35 mM LiCl + 35 mM NaCl as the external solutions. (c) Dialysis cassettes after dialysis, showing gel formation for LiCl, no gel for NaCl, and partial gelation for the mixed-salt condition.
